# Mapping the Hsp90 Genetic Interaction Network in *Candida albicans* Reveals Environmental Contingency and Rewired Circuitry

**DOI:** 10.1371/journal.pgen.1002562

**Published:** 2012-03-15

**Authors:** Stephanie Diezmann, Magali Michaut, Rebecca S. Shapiro, Gary D. Bader, Leah E. Cowen

**Affiliations:** 1 Department of Molecular Genetics, University of Toronto, Toronto, Canada; 2 The Donnelly Centre, University of Toronto, Toronto, Canada; Stanford University School of Medicine, United States of America

## Abstract

The molecular chaperone Hsp90 regulates the folding of diverse signal transducers in all eukaryotes, profoundly affecting cellular circuitry. In fungi, Hsp90 influences development, drug resistance, and evolution. Hsp90 interacts with ∼10% of the proteome in the model yeast *Saccharomyces cerevisiae*, while only two interactions have been identified in *Candida albicans*, the leading fungal pathogen of humans. Utilizing a chemical genomic approach, we mapped the *C. albicans* Hsp90 interaction network under diverse stress conditions. The chaperone network is environmentally contingent, and most of the 226 genetic interactors are important for growth only under specific conditions, suggesting that they operate downstream of Hsp90, as with the MAPK Hog1. Few interactors are important for growth in many environments, and these are poised to operate upstream of Hsp90, as with the protein kinase CK2 and the transcription factor Ahr1. We establish environmental contingency in the first chaperone network of a fungal pathogen, novel effectors upstream and downstream of Hsp90, and network rewiring over evolutionary time.

## Author Summary

Hsp90 is an essential and conserved molecular chaperone in eukaryotes that assists with folding diverse proteins, especially regulators of cellular signaling. By activating signaling in response to environmental cues, Hsp90 has a profound impact on myriad aspects of biology. In fungi, Hsp90 influences development, drug resistance, and evolution. In the model yeast *Saccharomyces cerevisiae*, Hsp90 interacts with ∼10% of proteins. In the leading human fungal pathogen, *Candida albicans*, only two interactions have been identified. We conducted a chemical genetic screen to elucidate the *C. albicans* Hsp90 interaction network under diverse stress conditions. The majority of the 226 genetic interactors are important for growth under specific conditions, suggesting that they act downstream of Hsp90 and that the network is environmentally contingent. For example, the kinase Hog1 depends upon Hsp90 for activation. Only a few interactors are important for growth in many conditions, suggesting that they act upstream of Hsp90. For example, the protein kinase CK2 regulates function of the Hsp90 chaperone machine and the transcription factor Ahr1 governs *HSP90* expression. Thus, we identify novel effectors upstream and downstream of Hsp90, and establish the first chaperone network of a fungal pathogen, with evidence for environmental contingency and network rewiring over evolutionary time.

## Introduction

Hsp90 is an essential and highly conserved molecular chaperone in all eukaryotes that specializes in folding metastable client proteins, many of which are signal transducers [1], [2]. Together with co-chaperones, Hsp90 interacts dynamically with client proteins regulating their stability and activation. Hsp90 function is subject to complex regulation by post-translational modification, including phosphorylation and acetylation, and depends upon an ATP binding and hydrolysis cycle [3], [4]. Hsp90 is generally expressed at much higher levels than required for basal function, however, environmental stress can induce global problems in protein folding and thereby overwhelm Hsp90's functional capacity [5]. As an environmentally contingent hub of protein homeostasis and regulatory circuitry, Hsp90 has profound effects on biology, disease, and evolution. Hsp90 modulates the phenotypic effects of genetic variation in an environmentally responsive manner [6], [7], [8], [9], influencing ∼20% of observed natural genetic variation and serving both to maintain phenotypic robustness and promote diversification [7].

Hsp90's broad influence on current genomes in part reflects its extensive connectivity in interaction networks and its profound impact on cellular circuitry. A global analysis of the Hsp90 chaperone network has thus far only been achieved in *S. cerevisiae*. Systematic proteomic and genomic methods have been applied to map physical, genetic, and chemical-genetic interactions, revealing that Hsp90 interacts with ∼10% of the proteome [10], [11]. In addition to identifying known co-chaperones and client proteins, these network analyses identified new Hsp90 client proteins as well as novel co-factors that link Hsp90 with chromatin remodeling and epigenetic gene regulation. A subsequent chemical-genetic screen identified distinct Hsp90 interactions at elevated temperatures, suggesting that specialized chaperone functions mediate responses to environmental stress [12]. While there are numerous conserved client proteins between *S. cerevisiae* and other eukaryotes, there is evidence for plasticity in the Hsp90 chaperone machine, with differences in co-chaperones even between *S. cerevisiae* and another model yeast, *Schizosaccharomyces pombe* [13]. The extent to which the Hsp90 chaperone network has been rewired over evolutionary time remains unknown.

Fungi provide not only the most powerful eukaryotic genetic model systems, but also a major threat to human health, and Hsp90 holds great promise as a therapeutic target [14], [15]. Invasive fungal infections are a leading cause of mortality among immunocompromised individuals, including those with cancer and HIV [16]. Treatment of fungal infections is hampered by the limited number of antifungal drugs, host toxicity, and the emergence of drug resistance [14], [17]. We previously established that Hsp90 regulates the emergence and maintenance of resistance to the most widely deployed classes of antifungal drugs in the clinic, the azoles and echinocandins [6], [15], [18], [19]. Compromising Hsp90 function can transform antifungals from ineffective to highly efficacious in combating otherwise lethal infections caused by the most prevalent fungal pathogens of humans, *Candida albicans* and *Aspergillus fumigatus* [15]. In *C. albicans*, Hsp90 regulates not only drug resistance, but also morphogenesis and virulence [20]. Despite the therapeutic potential of targeting Hsp90, mouse model studies revealed that Hsp90 inhibitors in clinical development as anti-cancer agents have toxicity in the context of an acute fungal infection [15], motivating the search for fungal-selective Hsp90 inhibitors and fungal-specific components of the Hsp90 chaperone network.

*C. albicans* provides the ideal fungal pathogen with which to dissect the Hsp90 chaperone network given its clinical relevance and its dependence on Hsp90 for drug resistance, virulence, and temperature-dependent morphogenesis. *Candida* species account for 88% of all hospital-acquired fungal infections [16]. *C. albicans* is the leading fungal pathogen of humans worldwide with mortality rates approaching 50%, and is the fourth most common cause of hospital-acquired infections [16], [21], [22]. Hsp90 regulates resistance to both azoles and echinocandins by stabilizing the protein phosphatase calcineurin and the terminal mitogen-activated protein kinase (MAPK) in the Pkc1 cell wall integrity pathway, Mkc1 [18], [19]. To date, these are the only two Hsp90 interactors identified in a fungal pathogen. Although genetic analyses with *C. albicans* have been hampered by its obligate diploid state and lack of a complete sexual cycle, recently homozygous mutant libraries have been developed to enable systematic screens and genomic analyses [23], [24], [25], [26].

Here, we mapped the first Hsp90 genetic interaction network in a fungal pathogen. We conducted a chemical-genetic screen with the first *C. albicans* homozygous transposon insertion mutant library containing 1,248 strains and covering ∼10% of the genome. Growth was scored under standard conditions as well as five stress conditions, both in the presence and absence of the Hsp90 inhibitor geldanamycin. Hypersensitivity to geldanamycin is indicative of an Hsp90 genetic interaction. The resulting network of interactions was extensively contingent on the environment. Most of the 226 genetic interactors were identified as important for growth only under specific conditions, suggesting that they operate downstream of Hsp90. Consistent with this model, Hsp90 depletion led to reduction in protein levels of several candidate interacting kinases, including Hog1 for which protein levels were reduced and stress-induced activation was abolished. Only a few genetic interactors were identified in many of the screens, and these likely operate upstream of Hsp90. Consistent with this model, interactors identified in five of the six screens include the regulatory subunits of casein kinase CK2, which governed function of the Hsp90 chaperone machine, and the transcription factor Ahr1, which promoted *HSP90* expression. The *C. albicans* Hsp90 genetic interaction network has been rewired relative to its *S. cerevisiae* counterpart, with a small but significant set of interactions conserved. Thus, we establish environmental contingency in the first Hsp90 chaperone network of a fungal pathogen, novel effectors upstream and downstream of Hsp90, and rewiring over evolutionary time.

## Results

### Chemical genetic screening reveals an environmentally contingent Hsp90 genetic interaction network

We conducted a chemical genetic screen employing a stationary liquid assay with the first *C. albicans* homozygous mutant library that covers ∼10% of the genome (661 genes) [23], [25]. To identify Hsp90 genetic interactors, the library was screened for mutants hypersensitive to pharmacological inhibition of Hsp90 with geldanamycin, which binds with high affinity to Hsp90's unusual ATP binding pocket and thereby blocks ATP-dependent chaperone function [27]. The screen was conducted under standard growth conditions (37°C), general stress conditions (elevated temperature of 41°C or osmotic stress exerted by sodium chloride (NaCl)), and specific stress conditions exerted by drugs targeting the endoplasmic reticulum (nucleoside antibiotic tunicamycin), the cell wall (echinocandin caspofungin), or the cell membrane (azole fluconazole) (Figure S1A, Table 1). Following incubation, growth was monitored by optical density and normalized relative to the geldanamycin-free control (Figure S1). Control strains with different geldanamycin sensitivities were included in each screen (Figure S1B).

**Table 1:** Hsp90 genetic interactions identified in six screens.

Stress condition	Screen	Incubation time (d)	Total number of interactions	Number of unique interactions	GO category enrichment (P-Value)
					
No stress	37°C	2	69	27	
					
General stress	41°C	4	64	32	Phosphorylation (0.005)
					Protein amino acid phosphorylation (0.005)
					Phosphorus metabolic process (0.024)
					Phosphate metabolic process (0.024)
					Regulation of cellular process (0.007)
					Regulation of biological process (0.03)
					
	0.55 M NaCl	3	27	5	SAGA complex (0.033)
					SAGA-like and SAGA-type complex (0.033)
					CK2 complex (0.033)
					UTP-C complex (0.033)
					Protein complex (0.015)
					Macromolecular complex (0.036)
					Post-translational modification (0.043)
					Nucleolar part (0.033)
					Drug response (0.001)
					
Specific stress	1 µg/ml	5	57	29	Cell cortex (0.04)
	Tunicamycin (T)				Protein complex (0)
					Protein amino acid phosphorylation (0)
					Protein modification process (0)
					Macromolecule modification (0)
					Macromolecular complex (0)
					Macromolecule metabolic process (0)
					Cellular macromolecule metabolic process (0)
					Phosphorylation (0)
					Phosphorus metabolic process (0.005)
					Phosphate metabolic process (0.005)
					Cellular protein metabolic process (0)
					Protein metabolic process (0)
					Post-translational protein modification (0)
					Drug response (0.001)
					Stimulus response (0.005)
					Chemical stimulus response (0.021)
					
	0.1 µg/ml	9	73	52	
	Caspofungin (C)				
					
	0.1 µg/ml	3	32	12	
	Fluconazole (F)				

**Figure 1:**
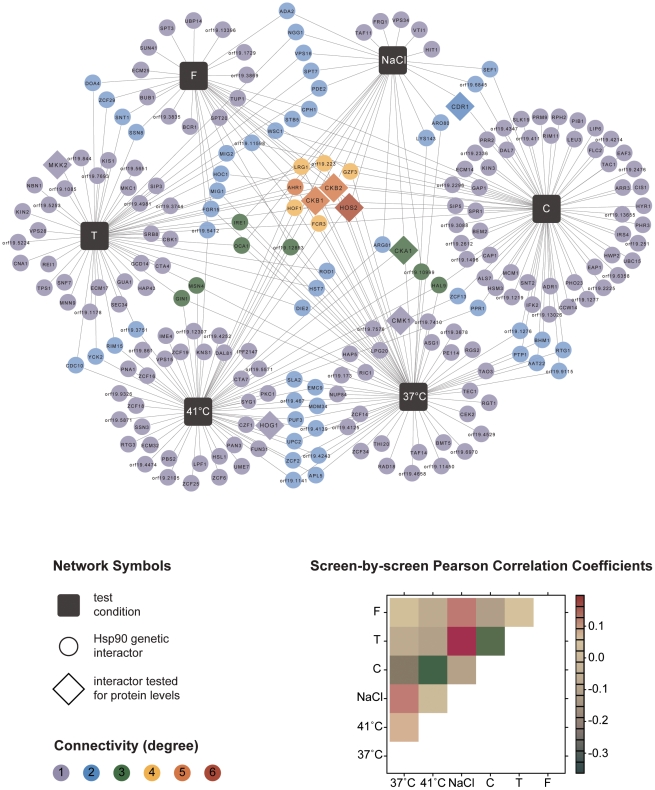
Global *C. albicans* Hsp90 genetic interaction network. The network comprises a total of 226 genetic interactions (circles, diamonds) as identified in six different experimental conditions (black boxes). In addition to interactions required for normal growth in RPMI (37°C), the network includes interactions identified during general stresses (41°C, NaCl) and specific stresses (tunicamycin (T), caspofungin (C), and fluconazole (F); Table 1). Each interaction is color-coded to reflect its degree of connectivity (frequency of occurrence in the six screens). Connectivity ranges from one (grey) to six (red). Every interactor is connected by edges to the one or more screens it had been identified in. An interaction that occurred multiple times has multiple edges. Eight interactors, with varying degrees of connectivity, were subsequently tested for protein levels upon Hsp90 depletion to determine if Hsp90 affects expression or stability of the interactor (diamonds). Pearson correlation coefficients are color-coded depending on the strength of the interaction (lower right).

The six screens yielded a total of 226 distinct Hsp90 genetic interactors (Figure 1 and Table S1). These were displayed as a network showing relationships between interactors and the screen conditions in which they were identified (Figure 1). The number of interactions differed widely between screens, as did the extent of overlap (Table 1). The cell wall stress screen (caspofungin) revealed the most interactions with 73 in total, 52 of which were unique; this screen was negatively correlated with all of the other screens (Figure 1), a unique pattern. Most genetic interactions identified were specific to one or two conditions, and very few were common to four or more conditions tested. Only nine interactions were identified in at least four screens; four of these were identified in at least five screens (*AHR1*, *CKB1*, *CKB2*, and *HOS2*) and only one in all six screens (*HOS2*).

**Figure 2:**
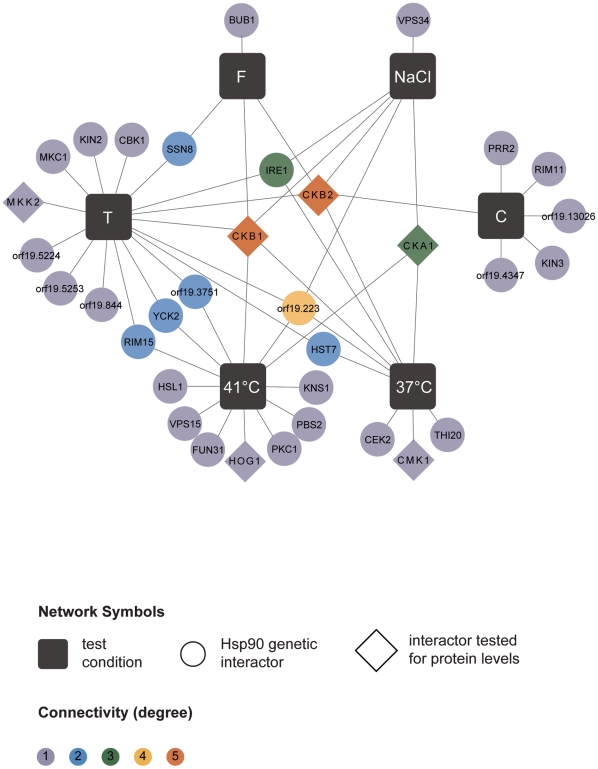
Hsp90 kinase genetic interaction network. Of the 226 interactions, 34 are with kinases. Kinases are color-coded depending on their degree of connectivity, ranging from grey for one connection to orange for five connections. Kinases and test conditions (black squares) are connected with each other via edges. While the caspofungin screen shared only one of its kinase interactors (*CKB2*) with another screen, every other screen shared half or more of its interactors with another screen. For six kinases (diamonds), protein levels were measured upon Hsp90 depletion.

Next, we tested the Hsp90 genetic interactors for enrichment of gene ontology (GO) gene function categories relative to the composition of the library. *C. albicans* Hsp90 genetic interactors were enriched for macromolecular complexes (*P* = 0.005), protein complexes (*P* = 0.005), protein modification processes (P = 0.018), biopolymer modification (P = 0.018), and post-translational protein modifications (*P* = 0.018). Kinases comprised 34 of the 226 Hsp90 genetic interactors identified (Figure 2), and were enriched from 10% of the library to 15% of the genetic interactors; 29 of the kinases were specific to one or two screens. The high temperature and tunicamycin screens identified the largest number of kinases, with seven each and three shared between them. Screen-specific GO enrichment varied (Table 1), consistent with the distinct suite of Hsp90 interactors identified as important for growth in the specific conditions.

Next, we examined whether our approach to map Hsp90 genetic interactions could also reveal proteins with functional dependence on Hsp90. Reassuringly, our screens identified both of the established *C. albicans* Hsp90 client proteins, Cna1 and Mkc1. Hsp90 physically interacts with and stabilizes the catalytic subunit of the protein phosphatase calcineurin, Cna1, such that depletion of Hsp90 leads to depletion of calcineurin, blocking calcineurin-dependent stress responses [19]. Hsp90 also stabilizes the MAPK Mkc1, such that depletion of Hsp90 leads to depletion of Mkc1, blocking downstream stress responses [18]. *CNA1* and *MKC1* were identified as Hsp90 interactors in our tunicamycin screen, consistent with their role in mediating responses to endoplasmic reticulum stress [28], [29].

**Figure 3:**
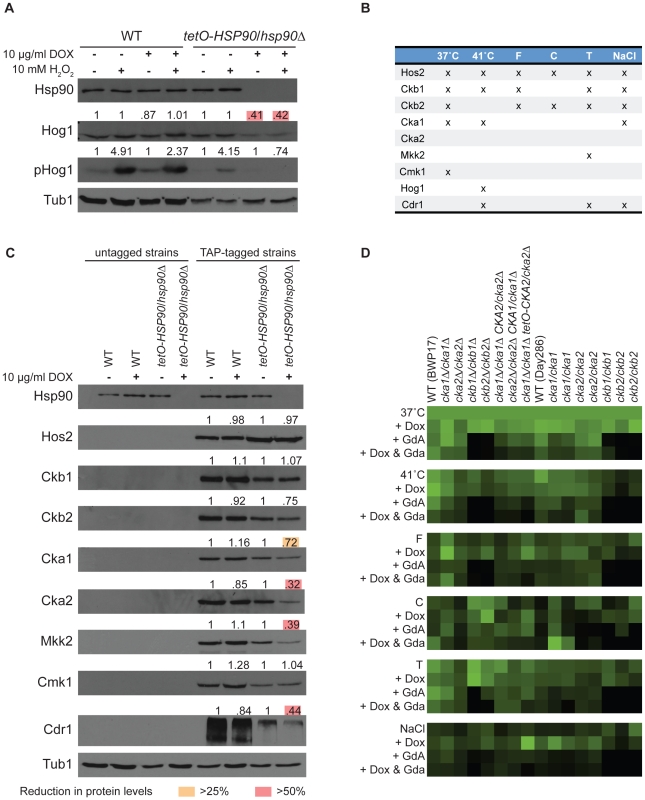
Analysis of high- and low-connectivity Hsp90 genetic interactors. (A) Hog1 (a low-connectivity interactor) protein levels and activation depend upon Hsp90. The wild type (WT, SN95) and *tetO-HSP90/hsp90*Δ (CaLC1411) strains were exposed to doxycycline, hydrogen peroxide (H_2_O_2_), or the combination, as indicated. Levels of Hsp90, Hog1, and activated phosphorylated Hog1 were monitored by Western analysis. Tubulin served as loading control. Protein levels were quantified, normalized to wild type and untreated controls and displayed above each blot. Hog1 levels were reduced by >50% (pink boxes) and activation was entirely abolished when Hsp90 was genetically compromised. (B) Nine candidate interactors, seven of which are kinases or kinase subunits, were selected for analysis of protein levels upon Hsp90 depletion based on their connectivity pattern in the global network. Three interactions occurred in five or six screens, and the remaining five occurred in three or less screens. (C) Three high-connectivity (Hos2, Ckb1, and Ckb2) and four low-connectivity (Cka1, Mkk2, Cmk1, and Cdr1) interactors, including six kinases or kinase subunits (Cka2), were tested for protein levels upon Hsp90 depletion. Relative protein levels are provided above each blot. High-connectivity interactors exhibit little dependence on Hsp90 with no reduction of protein levels by >25%. Low-connectivity interactors exhibit marked dependence on Hsp90, with reduction in proteins levels of >25% (yellow boxes) or even >50% (pink boxes) upon Hsp90 depletion. Tubulin served as loading control, and a representative blot is shown here. (D) Validation of screen results with CK2 transposon mutants, clean deletion mutants, and a catalytic subunit depletion strain. The deletion mutants phenocopy the transposon mutants, and doxycycline-mediated depletion of CK2 catalytic subunits confers hypersensitivity to geldanamycin in all six conditions.

To determine if we could identify novel functional dependence of a *C. albicans* protein on Hsp90 based on our dataset we turned to Hog1. Hog1 is a MAPK involved in osmoregulation that was identified as an Hsp90 interactor in our high temperature screen, and connections between Hog1 and Hsp90 have been established in other eukaryotes. In *S. cerevisiae*, Hog1 interacts with Hsp90 and co-chaperone Cdc37, which facilitates Hsp90's kinase specificity, and mutation of Cdc37 leads to reduced Hog1 levels and impaired downstream stress responses [30]. The mammalian homolog of Hog1, p38, is an Hsp90 client and interacts with Hsp90 via Cdc37 [31]; inhibition of Hsp90 leads to autoactivation of p38, suggesting that the Hsp90-Cdc37 complex functions as a negative regulator of p38 in mammalian cells as opposed to its role as a positive regulator in *S. cerevisiae*. We tested the impact of Hsp90 depletion on levels of both total Hog1 protein and activated dually phosphorylated Hog1 in response to osmotic stress induced by exposure to hydrogen peroxide (Figure 3A). To deplete Hsp90, we used a strain with its only *HSP90* allele driven by a doxycycline-repressible promoter (*tetO-HSP90/hsp90*Δ). In the presence of doxycycline, Hsp90 levels were depleted in the *tetO-HSP90/hsp90*Δ strain and not the wild type. Depletion of Hsp90 led to a ∼60% reduction in the levels of total Hog1 and abolished stress-induced Hog1 activation (Figure 3A). Transcript levels of *HOG1* were reduced by ∼30% upon Hsp90 depletion (Figure S2), suggesting that Hsp90 affects expression as well as activation, or stability of the activated form, of this MAPK. These results confirm that our chemical genetic screen can identify client proteins with functional dependence on Hsp90.

### Hsp90 affects stability or expression of low-connectivity genetic interactors

Given that our chemical genetic screens identify Hsp90 genetic interactions based on importance for growth under distinct environmental conditions, we hypothesized that low-connectivity interactors act downstream of Hsp90 to mediate specific responses, whereas high-connectivity interactors act upstream of Hsp90 to regulate its function or expression. To test this hypothesis, we determined the impact of Hsp90 depletion on three high- and five low-connectivity interactors (Figure 3B). The high-connectivity interactors (*HOS2*, *CKB1*, and *CKB2*) were identified in five or six conditions and the low-connectivity interactors (*CKA1*, *MKK2*, *CMK1*, *CDR1*, and *HOG1*) in up to three. With the exception of Hog1, which was discussed above, all candidate interactors were TAP-tagged to monitor protein levels upon Hsp90 depletion. Consistent with our hypothesis, depletion of Hsp90 caused greater reduction of protein levels for low-connectivity interactors than high-connectivity interactors (*P* = 0.0430); protein levels of four of the five low-connectivity interactors were reduced by greater than 25%, while none of the high-connectivity interactors exhibited this magnitude of reduction (Figure 3C). To distinguish more indirect effects on gene expression from effects on protein stability, we monitored transcript levels for all interactors that showed substantial reduction in protein levels. Of the five low-connectivity interactors tested, only one had significantly reduced transcript levels upon Hsp90 depletion, *HOG1* (Figure S2). Both established *C. albicans* client proteins that require Hsp90 for stability, Cna1 [19] and Mkc1 [18], were also low-connectivity interactors. These findings suggest that low-connectivity interactors depend on Hsp90 for stability or expression while high-connectivity interactors do not.

A striking observation was that two of the high- (*CKB1* and *CKB2*) and one of the low-connectivity interactors (*CKA1*) are subunits of protein kinase CK2. CK2 is a serine/threonine protein kinase and phosphorylates many substrates including yeast and human Hsp90, thereby regulating its function [32]. Like many kinases that phosphorylate Hsp90, CK2 is also an Hsp90 client in mammalian cells [33], suggesting that feedback loops might enable kinases to modulate their chaperoning and activation. In *C. albicans*, depletion of Hsp90 leads to reduced levels of both Cka1 and Cka2 catalytic subunits. Cka1 was identified as low-connectivity in our screens and Cka2 was not identified although it was present in the library (Figure 3B, 3C). Hsp90 depletion did lead to reduced levels of Cka2 protein (Figure 3C) and *CKA2* transcript (Figure S2). Protein levels of the two high-connectivity regulatory subunits, Ckb1 and Ckb2, remained relatively stable despite Hsp90 depletion (Figure 3C). These findings suggest that the catalytic CK2 subunits may be more dependent upon Hsp90 than the regulatory subunits, and that the regulatory subunits may act upstream to modify Hsp90 function. If the regulatory subunits function with the catalytic subunits to phosphorylate Hsp90, one would expect the catalytic subunits to have been high-connectivity interactors; that they were not could be due to their partial redundancy [34], [35]. To test this, we constructed a strain lacking Cka1 and in which Cka2 could be depleted by doxycycline-mediated transcriptional repression (*cka1*Δ/*cka1*Δ *tetO-CKA2/cka2*Δ, Figure S3). We repeated all six screens with the CK2 transposon mutants, clean deletion mutants, and our catalytic subunit depletion strain. The deletion mutants phenocopied the transposon mutants, confirming that the screens were reproducible and the original phenotypes of the transposon mutants were valid (Figure 3D). Further, depletion of the CK2 catalytic subunits conferred hypersensitivity to geldanamycin in all six screens (Figure 3D), confirming that the catalytic subunits would indeed have been high-connectivity interactors if not for their redundancy. Thus, we establish important functional connections between Hsp90 and CK2 in *C. albicans*.

### CK2 regulatory subunits affect phosphorylation of Hsp90 or Cdc37, or protein levels of Hsp90, Cdc37, and Hog1

**Figure 4:**
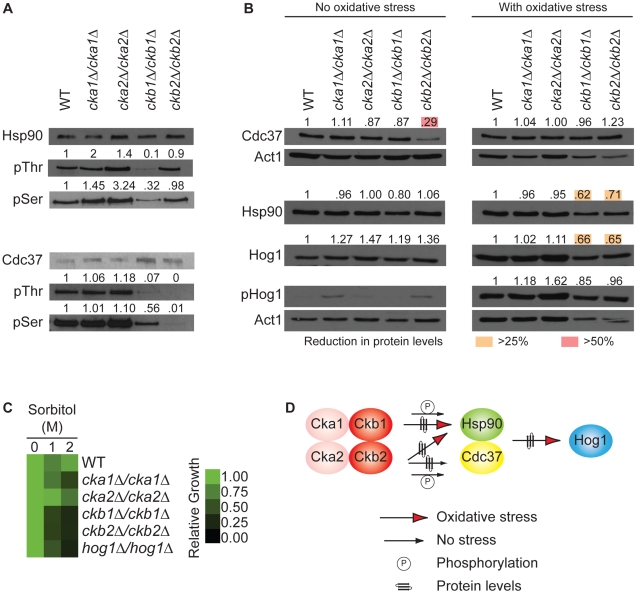
The protein kinase CK2 regulatory subunits regulate function of the Hsp90/Cdc37 protein complex. (A) Hsp90 serine and threonine phosphorylation is severely reduced in the *ckb1*Δ/*ckb1*Δ mutant, and Cdc37 serine and threonine phosphorylation is severely reduced in both the *ckb1*Δ/*ckb1*Δ and *ckb2*Δ/*ckb2*Δ mutants. Hsp90 or Cdc37 were immunoprecipitated and Western blots were hybridized with CaHsp90, TAP (to detect Cdc37-TAP), phosphothreonine, or phosphoserine antibodies. The ratio of phosphorylated to unphosphorylated Hsp90 or Cdc37 in each CK2 mutant was quantified relative to the wild type. (B) Western analysis demonstrates that Cdc37 levels are severely reduced (>50%, red box) in the mutant lacking the regulatory subunit Ckb2 (*ckb2*Δ/*ckb2*Δ) in the absence of external stress compared to the wild type (WT, BWP17); Hsp90 and Hog1 levels, however, are reduced (>25%, yellow box) in strains that lack the regulatory subunits (*ckb1*Δ/*ckb1*Δ or *ckb2*Δ/*ckb2*Δ) in response to oxidative stress in the form of a 10 minute treatment with 1 mM hydrogen peroxide. Actin served as loading control. (C) Deletion of CK2 regulatory subunits, *CKB1* or *CKB2*, phenocopies deletion of *HOG1* in terms of hypersensitivity to high osmolarity stress exerted by sorbitol. Growth is quantitatively displayed with color as indicated with the color bar. (D) Our results support a model in which the regulatory subunits of CK2 (Ckb1 and Ckb2) affect phosphorylation of Hsp90 and Cdc37, protein levels of the Hsp90-Cdc37 complex under basal or stress conditions, and levels of the target kinase Hog1.

Next, we tested whether CK2 phosphorylates threonine and serine residues of Hsp90 and Cdc37 in *C. albicans*. In *S. cerevisiae* Hsp90 is phosphorylated on at least 11 residues [36], including threonine 22 by CK2 [32]. CK2 also phosphorylates the Hsp90 co-chaperone Cdc37, which is critical for proper binding to kinases and for their stability [37]. To determine if CK2 phosphorylates Hsp90 and/or Cdc37 in *C. albicans*, we immunoprecipitated either Hsp90 or Cdc37 from the wild type and mutants lacking CK2 components and monitored levels of threonine and serine phosphorylation relative to total immunocprecipitated Hsp90 or Cdc37 protein. Threonine phosphorylation of Hsp90 was reduced by 90% in the *ckb1*Δ/*ckb1*Δ mutant and serine phosphorylation was reduced by 68% (Figure 4A). That phosphorylation was not reduced in the catalytic subunit mutants could be due to their partial redundancy [34], [35], consistent with our screen results (Figure 3D). That phosphorylation was not reduced in the *ckb2*Δ/*ckb2*Δ mutant might suggest that Ckb2 directs phosphorylation of fewer threonine or serine residues than Ckb1 under these conditions, or that it plays a more important role in phosphorylation of other targets. Consistent with the latter possibility, threonine and serine phosphorylation of Cdc37 was largely abolished in the *ckb2*Δ/*ckb2*Δ mutant (Figure 4A). Threonine phosphorylation of Cdc37 was also reduced by greater than 90% in the *ckb1*Δ/*ckb1*Δ mutant, while serine phosphorylation was reduced by 44% (Figure 4A). Complementation of the *ckb1*Δ/*ckb1*Δ and *ckb2*Δ/*ckb2*Δ mutants with wild-type alleles of *CKB1* or *CKB2* restored phosphorylation of Hsp90 and Cdc37 (Figure S4A and data not shown). Thus, *C. albicans* CK2 regulates serine and threonine phosphorylation of both Hsp90 and Cdc37.

Given that phosphorylation of Hsp90 [32] or its co-chaperone Cdc37 [37] can affect stability and function of target kinase client proteins in *S. cerevisiae*, we assessed the impact of deletion of each of the four *C. albicans* CK2 subunits on levels of Hsp90, Cdc37, and target kinase Hog1, as well as on Hog1 activation. Cells were grown in standard conditions or with a short burst of oxidative stress in order to monitor Hog1 activation via phosphorylation. Consistent with our hypothesis that the high-connectivity regulatory subunits of CK2 function upstream of Hsp90, protein levels of Hsp90, Cdc37, and Hog1 were reduced substantially in the *ckb1*Δ/*ckb1*Δ and *ckb2*Δ/*ckb2*Δ mutants (Figure 4B). Interestingly, there was a stress-dependent difference in the impact of CK2 subunit deletion. During standard growth conditions, the only change in proteins levels greater than 25% was that Cdc37 levels were reduced by 71% in the *ckb2*Δ/*ckb2*Δ mutant (Figure 4B, left panel). In response to oxidative stress, Hsp90 levels were reduced in both the *ckb1*Δ/*ckb1*Δ (38%) and *ckb2*Δ/*ckb2*Δ (29%) mutants (Figure 4B, right panel). Hog1 levels were also reduced in response to oxidative stress in both the *ckb1*Δ/*ckb1*Δ (34%) and *ckb2*Δ/*ckb2*Δ (35%) mutants (Figure 4B, right panel). Hog1 activation was not abolished (Figure 4B), unlike with depletion of Hsp90 (Figure 3A). Complementation of key mutants with a wild-type allele of *CKB1* or *CKB2* fully restored Hsp90 and Cdc37 protein levels, and partially restored Hog1 protein levels (Figure S4B), confirming that the observed effects are indeed due to the specific gene deletions. Thus, modification of the Hsp90-Cdc37 complex via the CK2 regulatory subunits is stress-dependent, such that Ckb2 is required for Cdc37 levels in the absence of stress, while Ckb1 and Ckb2 are both required for Hsp90 and Hog1 levels during oxidative stress.

If the regulatory subunits of CK2 regulate function of the Hsp90-Cdc37 complex and downstream clients such as Hog1, then one would predict that deletion of these CK2 subunits would phenocopy deletion of Hog1. To test this, we monitored growth during osmotic stress exerted by sorbitol, given Hog1's importance for osmotic stress responses. We found that the *ckb1*Δ/*ckb1*Δ, *ckb2*Δ/*ckb2*Δ and *hog1*Δ/*hog1*Δ mutants were equally hypersensitive to high osmolarity (Figure 4C). Complementation of the mutants with a wild-type allele of *CKB1*, *CKB2*, or *HOG1* restored high osmolarity growth, confirming that the phenotypes observed are a consequence of the specific gene deletions (Figure S4C). Taken together, our results support the model that function of the Hsp90-Cdc37 chaperone complex is modulated in a stress-dependent manner by the high-connectivity interactors Ckb1 and Ckb2, thereby affecting target kinases (Figure 4D).

### ∼17% of the *C. albicans* Hsp90 genetic interactions are conserved in *S. cerevisiae*

To date, the most extensive studies of the Hsp90 chaperone network have been carried out in *S. cerevisiae* [11], [12]. Chaperone networks have been examined in the protozoan parasite *Plasmodium falciparum* [38], but comparative analysis was limited due to high protein interaction network divergence from other eukaryotes [39], [40]. As of yet, comparative analysis of Hsp90 genetic interaction networks has not been feasible due to the lack of large-scale interaction data in species other than *S. cerevisiae*.

Comparison of our *C. albicans* Hsp90 genetic interaction set with those from *S. cerevisiae* genetic screens [11], [12] revealed a small but significant overlap (Hypergeometric, *P* = 0.004), despite their highly similar co-chaperone machineries (Figure S5). The *C. albicans* library screened contains insertions in 428 genes that have homologs in *S. cerevisiae*, 59 of which are genetic interactors in *S. cerevisiae*; of these 59, 30 were unique to *S. cerevisiae* (Figure S5). The 29 Hsp90 interactors that were conserved out of 171 *C. albicans* Hsp90 interactors that have a *S. cerevisiae* homolog indicate that only ∼17% of *C. albicans* Hsp90 genetic interactions are conserved. Thus, the chaperone network has been rewired considerably over evolutionary time.

The conserved interactors were distributed throughout the *C. albicans* Hsp90 network but differences in the extent of conservation were observed in different stress conditions. While ∼25% of Hsp90 genetic interactions identified in the cell membrane stress screen (fluconazole) were conserved, less than 10% of the those from the cell wall stress screen (caspofungin) were conserved (Figure S5, left insert). Despite the low level of conservation, both *S. cerevisiae* and *C. albicans* networks exhibited similar responses to elevated temperature in that they both maintained a large fraction of their interactions (roughly a half [12] and a third, respectively) (Figure S5, right insert). While the particular interactions involved may differ, similar proportions of the genome remains associated with Hsp90 during high temperature growth in *C. albicans* and *S. cerevisiae*.

We tested whether the conserved Hsp90 genetic interactors might reflect dependence of the corresponding proteins on Hsp90. We monitored the impact of Hsp90 depletion on protein and transcript levels of three conserved genetic interactors (*CKB2*, *CKA1*, and *CDR1*). Cka1 protein levels were reduced by >25% and Cdr1 levels by >50% upon depletion of Hsp90 (Figure 3), and transcript levels of both *CKA1* and *CDR1* remained unchanged, suggesting that some but not all of the conserved Hsp90 genetic interactions may also reflect a physical interaction.

### A novel high-connectivity interactor affects *HSP90* expression and morphogenesis

**Figure 5:**
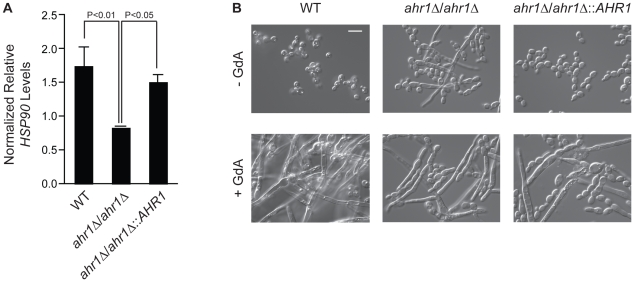
The high-connectivity interactor Ahr1 influences *HSP90* expression and morphogenesis. (A) *HSP90* transcript levels are reduced in the *ahr1*Δ/*ahr1*Δ mutant. *HSP90* transcript levels were measured in the wild type (SN152), the *ahr1*Δ/*ahr1*Δ mutant, and the *AHR1* complemented strain by quantitative RT-PCR and normalized to *GPD1*. Data are means ± standard deviation for triplicate samples. (B) The *ahr1*Δ/*ahr1*Δ mutant filaments in rich medium at 30°C, consistent with the effects of compromised Hsp90 function. Differential Interference Contrast microscopy of strains incubated in rich medium at 30°C for 24 hours with or without 10 µM geldanamycin (GdA). Scale bar is 10 µm.

Given the considerable rewiring of the Hsp90 chaperone network, we sought to characterize a novel Hsp90 interactor in *C. albicans*. We focused on *AHR1*, as it was identified as an Hsp90 genetic interactor in five out of our six screens. This zinc finger transcription factor binds to target promoters to regulate transcription of genes involved in adherence, morphogenesis, and virulence [24], [41]. Consistent with the expectation that high-connectivity interactors function upstream of Hsp90, we found that *HSP90* transcript levels were reduced in an *ahr1Δ/ahr1*Δ mutant (Figure 5A, t-test, *P* = 0.0298). Strikingly, deletion of *AHR1* phenocopies compromise of Hsp90 function leading to filamentation in rich medium at 30°C, canonical conditions for yeast growth (Figure 5B). Complementation with a wild-type allele of *AHR1* restores wild-type *HSP90* transcript levels and morphology (Figure 5). Thus, Ahr1 is a novel high-connectivity *C. albicans* Hsp90 interactor that influences *HSP90* expression and morphogenesis, a trait of central importance for virulence.

## Discussion

**Figure 6:**
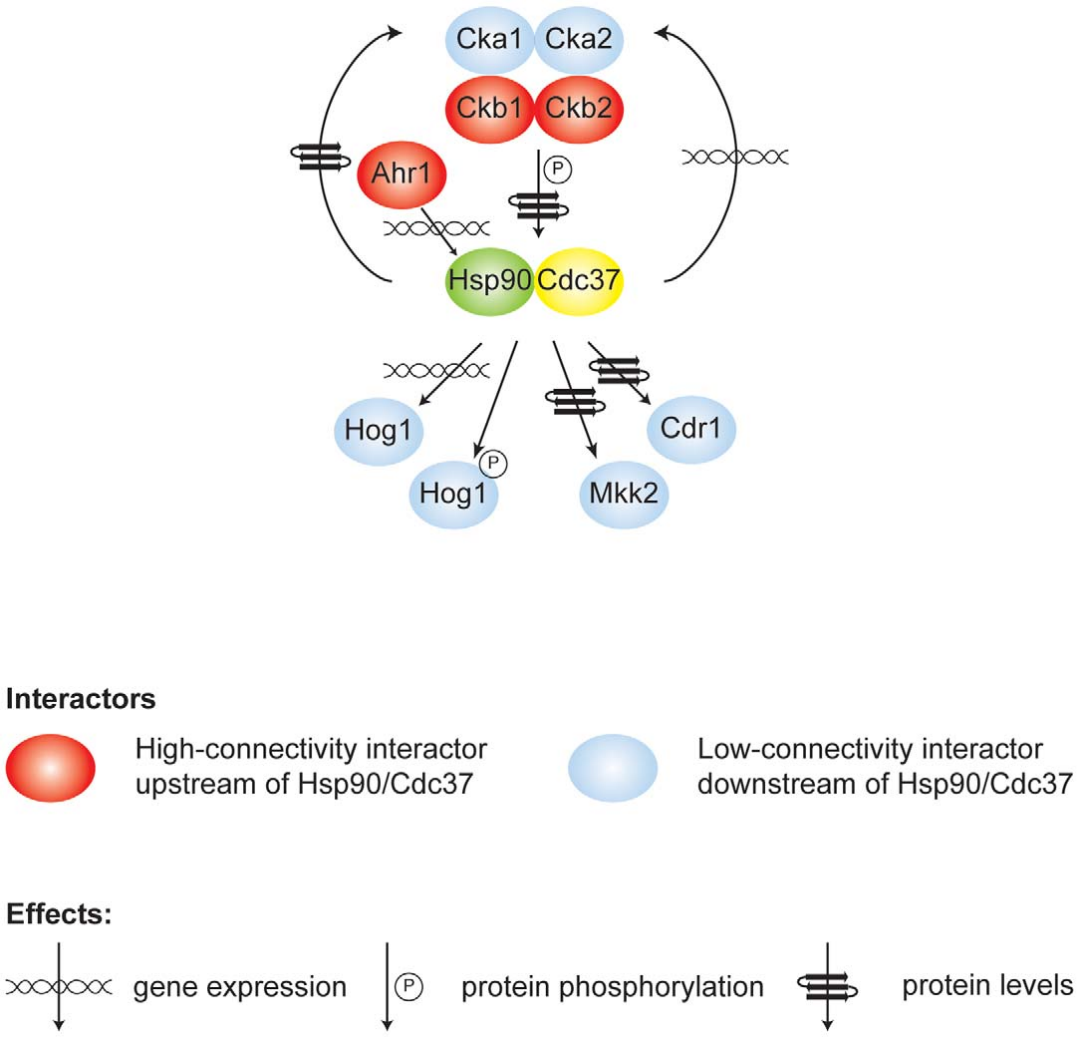
Model of high- and low-connectivity interactors identified in this screen that either modify the Hsp90/Cdc37 complex or are affected by it. The high connectivity interactors (red) modulate gene expression, protein levels, or phosphorylation of Hsp90 and Cdc37, while the chaperone complex regulates gene expression, protein levels or activation of low-connectivity interactors (blue).

Our results establish the first Hsp90 chaperone network of a fungal pathogen, novel effectors upstream and downstream of Hsp90, environmental contingency in the network, and network rewiring over evolutionary time. Based on our chemical genetic screen with the first *C. albicans* homozygous transposon insertion mutant library [23], [25], Hsp90 interacts with ∼4% of the genome (Figure 1), including many kinases (Figure 2). The proportion of the genome that interacts with Hsp90 is expected to increase upon screening additional mutants or stress conditions. The chaperone network is environmentally contingent, and most of the 226 genetic interactors are important for growth only under specific conditions, suggesting that they operate downstream of Hsp90, as with Hog1 (Figure 3A and Figure 6). Few genetic interactors are important for growth in many environments, and these are poised to operate upstream of Hsp90 to regulate its function or expression, as with the protein kinase CK2 and transcription factor Ahr1 (Figure 4, Figure 5, and Figure 6). The *C. albicans* Hsp90 genetic interaction network is rewired relative to its *S. cerevisiae* counterpart (Figure S5), emphasizing the importance of dissecting the chaperone network in the pathogen to elucidate circuitry through which Hsp90 regulates key traits important for virulence.

Our study provides the first unbiased analysis of *C. albicans* Hsp90 interactors, and a glimpse of the circuitry through which Hsp90 governs drug resistance, morphogenesis, and virulence. Prior to this work, only two Hsp90 interactors were identified in *C. albicans*, the protein phosphatase calcineurin [19] and MAPK Mkc1 [18]. Both are Hsp90 genetic interactors in our tunicamycin screen (Figure 1 and Figure 2), consistent with their role in mediating responses to endoplasmic reticulum stress [28], [29], and validating that the genetic interactors we identify here also include physical interactors with functional dependence on Hsp90. The MAPK Hog1, an Hsp90 genetic interactor in our high temperature growth screen (Figure 1 and Figure 2), has been previously connected with Hsp90 in other eukaryotes. In *S. cerevisiae* and mammalian cells, Hog1/p38 interacts with Hsp90 via the co-chaperone Cdc37. In *S. cerevisiae*, Hog1 levels decrease upon compromising Hsp90-Cdc37 function and canonical (stress-induced) levels of Hog1 phosphorylation are reduced by ∼20% [30]. In mammalian cells, Hsp90-Cdc37 is dispensable for canonical activation of p38 and inhibition of Hsp90 leads to auto-activation of p38 [31]. In *C. albicans*, depletion of Hsp90 reduces Hog1 protein by ∼60% and abolishes stress-induced Hog1 activation (Figure 3A), suggesting that regulation of Hog1 activation in *C. albicans* is similar to that in *S. cerevisiae* but perhaps more dependent upon Hsp90. Hog1 is itself a global regulator of the *C. albicans* proteome induced in response to diverse stresses [42], and mutants lacking Hog1 display hypersensitivity to stress, altered morphogenesis, attenuated virulence in mouse models of systemic disease, and enhanced vulnerability to killing by phagocytes [43], [44]. Our identification of Ahr1 as a novel regulator of *HSP90* expression and morphogenesis (Figure 5) further validates that our chaperone network reveals novel regulators through which Hsp90 governs stress response, drug resistance, morphogenesis, and virulence.

There are distinct sets of Hsp90 genetic interactions under different conditions, establishing that the network is environmentally contingent and suggesting specialized Hsp90 functions in mediating responses to specific stresses. We reasoned that interactors that are low connectivity in the network, identified in only one to three screens, likely function downstream of Hsp90 to regulate cellular processes important for growth in specific environments. Indeed, depletion of Hsp90 causes greater reduction of protein levels for low-connectivity than high-connectivity interactors; protein levels for four (Hog1, Cka1, Mkk2, and Cdr1) out of the five low-connectivity interactors tested are reduced by greater than 25% (Figure 3C); out of these four only one showed a significant reduction in transcript levels (Figure S2), suggesting that low-connectivity interactors depend upon Hsp90 for stability or expression. The level of reduction of low-connectivity interactors upon Hsp90 depletion ranges from 28% to 61%, suggesting that additional factors contribute to their stability. The one low-connectivity interactor that does not show reduced protein levels upon Hsp90 depletion, a calmodulin-dependent kinase Cmk1, could still rely on Hsp90 for activation rather than stability, consistent with the finding that Cmk1 interacts with Hsp90 in the fungal pathogen *Sporothrix schenckii* [45], or alternatively could function in a pathway with which Hsp90 interacts. Thus, connectivity in the network can reveal functional properties of Hsp90 interactors in terms of the environmental conditions for which they enable adaptive responses.

Interactors that are high connectivity in the network are likely to function upstream of Hsp90 and thereby regulate its function or expression, impacting on growth in diverse conditions. None of the three high-connectivity interactors tested (Hos2, Ckb1, and Ckb2) show reduced proteins levels upon Hsp90 depletion (Figure 3C), consistent with the hypothesis that they function upstream of Hsp90. Since three out of the four protein kinase CK2 subunits interact genetically with Hsp90 in our screens, we tested the hypothesis that high-connectivity interactors regulate Hsp90 function by focusing on the high-connectivity CK2 regulatory subunits, Ckb1 and Ckb2. CK2 phosphorylates many cellular targets, including a conserved threonine in Hsp90 of *S. cerevisiae* (T22) and mammalian cells (T36 in hHsp90α) [32]. CK2-dependent phosphorylation of Hsp90 modulates chaperone activity, affecting the stability and function of diverse clients. CK2 also phosphorylates Cdc37, a prerequisite for proper binding to kinases and for their stability [37]. There is feedback such the Hsp90-Cdc37 chaperone also binds CK2 thereby promoting its stability and activation [33]. We provide the first evidence for a functional relationship between CK2 and Hsp90 or Cdc37 in *C. albicans*. Hsp90 serine and threonine phosphorylation is dramatically reduced in the *ckb1*Δ/*ckb1*Δ mutant (Figure 4). Cdc37 serine and threonine phosphorylation is also reduced in the *ckb1*Δ/*ckb1*Δ mutant, and is largely abolished in the *ckb2*Δ/*ckb2*Δ mutant (Figure 4). Redundancy of the CK2 catalytic subunits could explain why Hsp90 phosphorylation was not reduced in these mutants, consistent with our screen results (Figure 3D). Cdc37 levels depend on Ckb2 during standard growth, while Hsp90 levels depend on both Ckb1 and Ckb2 during oxidative stress (Figure 4). The *ckb1*Δ/*ckb1*Δ and *ckb2*Δ/*ckb2*Δ mutants have reduced levels of the Hsp90-Cdc37 target kinase Hog1 and phenocopy a *hog1*Δ/*hog1*Δ mutant in terms of hypersensitivity to oxidative stress (Figure 4), supporting the model that the high-connectivity CK2 regulatory subunits influence Hsp90-Cdc37 function. Taken together, our study reveals CK2 as the first regulator of *C. albicans* Hsp90 function and suggests that additional high connectivity interactors might also serve to regulate Hsp90 function or expression, as with Ahr1 (Figure 5).

Although Hsp90 is highly conserved, the Hsp90 chaperone network has been rewired over evolutionary time. Akin to the *S. cerevisiae* Hsp90 network, the *C. albicans* network prominently featured kinases, which were enriched from 10% in the library to 15% in the network. Enrichment for transcription factors [11], however, was not detected in *C. albicans*, despite transcription factors being well represented in the library. This may be due to network rewiring since the species diverged, as only ∼17% of the genetic interaction network is conserved (Figure S5), suggesting network remodeling during adaptation to specific ecological niches. Rewiring in response to selective pressure could also explain the large set of 73 Hsp90 interactors important for growth during cell wall stress (caspofungin), 52 of which are specialized for that stress, given our tested set of conditions (Figure 1, Table 1). Notably, Hsp90 governs survival in response to echinocandin-induced cellular stress in *C. albicans*, but not in *S. cerevisiae* [19]. Further, signaling pathways governing cell wall integrity pathways have been rewired between *C. albicans* and *S. cerevisiae* [23]. The fungal cell wall is essential for viability of fungal cells and is an elaborate structure, components of which are recognized by the vigilant cadre of immune cells in the human host [46]. As a commensal and opportunistic pathogen, *C. albicans* is likely to harbor circuitry orchestrating cell wall structure that was subject to strong selection in response to challenge by the host immune system. Indeed *C. albicans* can evade immune recognition and attack by masking its β-glucan [47]. The finding of evolutionary reconfiguration of the Hsp90 chaperone network in a fungal pathogen motivates future studies to map the chaperone network in diverse eukaryotic pathogens in which Hsp90 has been implicated in governing drug resistance, development, or virulence, such as the fungal pathogen *Aspergillus fumigatus* and the protozoan parasites *Plasmodium falciparum* and *Trypanosoma evansi* [15], [48]. Identifying pathogen-specific components of the Hsp90 chaperone network offers great therapeutic potential for the development of inhibitors to minimize host toxicity and cripple diverse eukaryotic pathogens.

## Materials and Methods

### Chemical genetic screen

The *C. albicans* transposon insertion mutant library was generously provided by Aaron Mitchell (Carnegie Mellon University) with additional plates obtained from the Fungal Genetics Stock Center and pinned onto YPD agar plates (1% yeast extract, 2% peptone, 2% dextrose, 2% agar). Strains were inoculated in 100 µl RPMI-1640 pH 7 (10.4 g/l RPMI-1640, 3.5% MOPS, 2% glucose, 20 mg/ml histidine, 80 mg/ml uridine), sealed with Adhesive Plate Seals (Thermo Scientific) and incubated overnight at 37°C while shaking at 200 rpm. Cells were then diluted twice. First, 1∶1,000 using the VP 408 96 Pin Multi-Blot Replicator (VP Scientific) in 1× phosphate buffered saline (PBS). Second, the PBS – *Candida* mixture was diluted 1∶10 in a total volume of 200 µl RPMI-1640, RPMI with 3 µM geldanamycin, RPMI with stressor (Table 1), and RPMI with 3 µM geldanamycin and stressor in flat bottom 96-well plates. Plates were incubated at 37°C for between two and nine days, depending on the stressor (Table 1). Following incubation, optical densities (ODs) were measured at λ = 600 nm.

ODs were recorded for RPMI alone, RPMI with geldanamycin, RPMI with stressor, and RPMI with geldanamycin and stressor, and normalized. The normalized values were transformed into heat maps, which represent growth as a function of color using Java TreeView 1.1.3. [49]. For each library plate, the RPMI alone was compared with the geldanamycin and the stress alone plates and the combination of both. A genetic interactor was defined as a mutant that responded with a severe growth defect or death to the combination of geldanamycin and stressor when neither geldanamycin alone nor the stressor alone impaired growth of the mutant. Genetic interactors were scored depending on the number of mutants available for a particular ORF: ‘1→0’ indicates that only one mutant was available and that mutant was severely hypersensitive to geldanamycin; ‘2→0’ indicates that both available mutants were severely hypersensitive to geldanamycin; and ‘1/2→0’ indicates that one of two available mutants was severely hypersensitive to geldanamycin with no growth and the other had impaired growth in the presence of geldanamycin compared to the wild-type strain Day 286. In the rare cases that more than two mutants for a particular gene were present in the library, at least two mutants had to exhibit severe hypersensitivity to geldanamycin to be scored as a genetic interaction.

### Network analyses and GO category enrichment

Networks of 226 global interactions and 34 kinase interactions were visualized with Cytoscape [50] and the layout manually improved for readability and clarity. A Fisher's Exact Test followed by a correction for multiple testing (empirical resampling) was used to identify GO terms that were enriched in the complete data set, in the different screens, and in the genetic interaction sets that were either unique to *C. albicans*, to *S. cerevisiae*, or shared by both. The GO enrichment analysis was performed with FuncAssociate [51] (download date April 13, 2011) using the program's default parameters on *C. albicans* gene lists and GO terms. All *C. albicans*-specific analyses were performed against a background of 661 genes in the library. When comparing *C. albicans* with *S. cerevisiae*, gene orthology information was obtained from the *Candida* Genome Database (http://www.candidagenome.org/).

### Strain and plasmid construction

All strains used here that were not part of the library (Table S2) were maintained in cryo-culture at −80°C in 25% glycerol. Genes of candidate interactors were tagged with the tandem affinity purification (TAP) tag [52] in the wild-type strain SN95 and its derivative CaLC1411 using a PCR-based strategy [53]. The tag and a selectable marker (*ARG4*) were PCR amplified from pLC573 (pFA-TAP-*ARG4* [53]), 200 to 400 µl of PCR product were ethanol precipitated, dissolved in 50 µl sterile water and transformed into *C. albicans* using standard protocols. Oligonucleotides used in this study are listed in Table S3. Correct genomic integration was verified using appropriate primer pairs that anneal ∼500 bp up or down stream from both insertion junctions together with primers oLC1593 (TAP-R) and oLC1594 (ARG4-F) that target the TAP tag and the selectable marker. The same TAP tagging strategy using pLC572 (pFA-TAP-*HIS1* [53]) was employed to tag *CDC37* and *HSP90* in the CK2 subunit deletion mutants. Details regarding TAP tagging of *HOS2*, complementation of CK2 subunits, and required strains and plasmids can be found in Text S1.

### Protein extraction, immunoprecipitation, and immune blot analysis

*C. albicans* CaLC239 (SN95) and CaLC1411 (*tetO-HSP90-hsp90*Δ) with and without TAP tagged interactors (Table S2) were grown overnight at 37°C in RPMI-1640 while shaking at 200 rpm. Stationary phase cultures were split, adjusted to an OD_600_ of 0.2 and one culture was treated with 10 µg/ml doxycycline (BD Biosciences), while the other was left untreated. After 24 hours of incubation at 37°C in RPMI-1640, cultures were re-adjusted to OD_600_ of 0.2 with and without doxycycline and grown to mid-log phase (OD_600_ 0.6–0.8). Between 15 and 50 ml were harvested from each culture, centrifuged for 10 minutes at 3000 rpm at 4°C, and washed once with ice-cold 1×PBS. Pellets were resuspended in 200 µl lysis buffer (50 mM Hepes pH 7.5, 150 mM NaCl, 5 mM EDTA, 1% Triton X100, protease inhibitor cocktail (Roche Diagnostics)) together with acid-washed glass beads and cells were mechanically disrupted by bead-beating for 3 minutes. To test for Hog1 activation via phosphorylation, proteins were extracted as described by LaFayette et al. [18]. Whole cell protein samples were diluted 1∶10 in water and subjected to Bradford analysis to determine protein concentrations. For separation by 8% or 10% SDS-PAGE, protein concentrations were adjusted in 6× Laemmli buffer and lysis buffer, between 1 µg and 50 µg loaded (Table S4) and separated at constant 120 Volts. Tub1 and Act1 served as loading controls. Details on antibodies used are provided in Table S4. To purify Hsp90 and Cdc37, Hsp90-TAP and Cdc37-TAP were immunoprecipitated with anti-IgG agarose as described by Singh et al. [19], with two modifications: phosphatase inhibitors were added to the lysis buffer (PhosStop, Roche Diagnostics) and protein samples were incubated with IgG-agarose for 2.5 hours at 4°C. After the final wash, 40 µl of 2× Laemmli buffer was added and 3 µl and 30 µl of the protein samples were separated by 10% SDS-PAGE for hybridization with CaHsp90, anti-TAP, PhosphoThreonine Q7, and PhosphoSerine Q5 antibodies, respectively.

Following separation, proteins were wet-transferred to a PVDF membrane (Bio-Rad Laboratories, Inc.) over night at 4°C and 30 V. Cdr1 was transferred for an additional hour at 100 V. Membranes were blocked for one hour in 1×PBS-T (1×PBS with 0.1% Tween 20 with 5% skimmed milk, washed for 5 minutes in 1×PBS-T and probed with the respective antibody. Primary antibodies were dissolved in 1×PBS-T, 2.5% skimmed milk and 0.003% sodium azide. All primary antibodies, except p38 MAPK and PhosphoThreonine Q7, and PhosphoSerine Q5, were left on the membrane for one hour at room temperature. p38, PhosphoThreonine, and PhosphoSerine antibodies were incubated over night at 4°C. Primary antibodies were washed off twice with 1× PBS-T for ten minutes and the membrane probed for one hour with secondary antibody, dissolved in 1× PBS-T and 5% milk. The secondary antibody was washed off twice with 1× PBS-T for five minutes and once with 1× PBS for five minutes. PhosphoThreonine Q7 and PhosphoSerine Q5 hybridizations were conducted according to the manufacturers instructions. Following exposure and development, films were scanned and protein levels compared using ImageJ (http://imagej.nih.gov/ij/index.html).

### RT–PCR

To monitor gene expression changes in response to *HSP90* depletion, strains SN95, CaLC1411, SN152, CaLC2114, and CaLC2115 were cultured as described above in preparation for protein extraction. To measure *CKA1* and *CKA2* expression levels, overnight cultures were diluted to an OD_600_ of 0.2 with or without 20 µg/ml doxycycline, grown for 24 hours and diluted again and cultured to mid-log phase. Upon reaching mid-log phase, RNA was then isolated using the QIAGEN RNeasy kit and cDNA synthesis was performed using the AffinityScript cDNA synthesis kit (Stratagene). PCR was carried out using the SYBR Green JumpStart Taq ReadyMix (Sigma-Aldrich) with the following cycle conditions: 94°C for 2 minutes, and 94°C for 15 seconds, 60°C for 1 minute, 72°C for 1 minute, for 40 cycles. All reactions were done in triplicate using the following primer pairs: *GPD1* (oLC752/753), *HSP90* (oLC754/755), *HOG1* (oLC1968/1969), *CKA1* (oLC1964/1965), *CKA2* (oLC1966/1967), *MKK2* (oLC1970/1971), *CDR1* (oLC1972/1973) (Table S3). Data were analyzed in the StepOne analysis software (Applied Biosystems).

### Morphogenesis assay and microscopy

Strains SN152 (WT), CaLC2114 (*ahr1*Δ/*ahr1*Δ) and CaLC2115 (*ahr1Δ/ahr1Δ::AHR1*) were grown for 24 hours in YPD at 30°C with and without 10 µM GdA while shaking at 200 rpm. Cells were then imaged using Differential Interference Contrast microscopy using a Zeiss Axio Imager.MI microscope and images analyzed with Axiovision software (Carl Zeiss, Inc.).

### Statistical analyses

To determine if protein levels differed significantly between high- and low-connectivity interactors, an unpaired t-test with Welch's correction was carried out. Expression level differences in low-connectivity interactors in response to Hsp90 depletion were evaluated with a one-way ANOVA with Bonferroni correction. Differences in *HSP90* expression levels were assessed using paired t-tests. All analyses were done using GraphPad Prism 4.0.

## Supporting Information

Figure S1 The homozygous transposon insertion mutant library composition and screen set up. (A) The majority of the transposon insertion mutant library is capable of growth during the different stress conditions tested, such that the library composition remains stable over screens. The majority of genes are represented by either one or two independent insertion mutants. (B) A color-coded example library plate. Each well is colored according to how many mutants are available for the relevant ORF. This plate contains a mixture of genes that are represented by one, two, or more than two mutants, as indicated by the color key on the right. To illustrate differences in growth that were observed during the screen, the photograph shows the control strains, included in each experiment, growing with and without geldanamycin. Wells were photographed after 48 hours of incubation at 37°C, and reduced growth in response to geldanamycin can be clearly seen in columns 3, 4, and 6. (C) The heat map was generated by normalizing ODs from strains grown with geldanamycin to those grown without. Duller shades of green indicate reduced growth and black represents lack of growth. A gene was considered a genetic interactor if: (i) the one available mutant was hypersensitive to geldanamycin (grey square); (ii) both available mutants were hypersensitive to geldanamycin (yellow rectangle); (iii) only one of two mutants showed no growth in response to geldanamycin (blue rectangle); or (iv) at least two of more available mutants were hypersensitive to geldanamycin.
(PDF)

Figure S2 Expression levels of low-connectivity interactor genes and *CKA2* in the wild type (SN95) and *tetO-HSP90/hsp90*Δ (CaLC1411) strains in response to depletion of Hsp90. Shown are the mean of three technical replicates and the standard deviation for each gene. *P*-values indicate statistical significance as calculated in a one-way ANOVA analysis.
(PDF)

Figure S3 Expression levels of *CKA1* (top panel) and *CKA2* (bottom panel) in the wild type (BWP17), deletion mutants, and the *tetO-CKA2/cka2*Δ strain. The wild type and the *tetO-CKA2/cka2*Δ strain were additionally treated with 20 µg/ml doxycycline.
(PDF)

Figure S4 Complementation of CK2 regulatory subunits restores Hsp90 threonine phosphorylation, as well as Hsp90, Cdc37, and Hog1 protein levels. (A) Hsp90 threonine phosphorylation is reduced in the *ckb1*Δ/*ckb1*Δ mutant but restored in the *ckb1*Δ/*ckb1*Δ*::CKB1* complementation strain. (B) Complementation of *CKB1* and *CKB2* fully restores Hsp90 and Cdc37 protein levels, and partially restores Hog1 levels. (C) Growth in 1 M sorbitol is restored upon complementation of *CKB1*, *CKB2*, or *HOG1*. Top row represents growth in the absence of sortbitol, and bottom row represents growth in the presence of sorbitol. Data were analyzed as in Figure 4C.
(PDF)

Figure S5 Hsp90 genetic interactions shared between *C. albicans* and *S. cerevisiae*. *C. albicans* Hsp90 genetic interactors that have a homolog in *S. cerevisiae* (blue) and have been shown to genetically interact with Hsp90 in *S. cerevisiae* (red) are mapped onto the global network. Of the high connectivity interactors, only *CKB1* is shared between both species. A quarter of the *C. albicans* genetic interactions identified in the fluconazole screen are shared with *S. cerevisiae* (left insert), while less than 10% of the caspofungin interactions are shared. Temperature-dependent genetic interaction profiles are similar between both species (right insert). About a third of the genetic interactions from the 37°C screen (standard *C. albicans* growth temperature) are maintained at elevated temperature (41°C) and close to half of the genetic interactions from the 30°C *S. cerevisiae* screen are maintained at elevated temperatures (37°C).
(PDF)

Table S1 Hsp90 genetic interactors.
(XLS)

Table S2 Strains used in this study.
(DOC)

Table S3 Oligonucleotides used in this study.
(DOC)

Table S4 Antibodies and conditions used in this study.
(DOC)

Text S1 Supporting Materials and Methods.
(DOC)
