# Supplementary figures and images for: Mapping the Hsp90 Genetic Interaction Network in Candida albicans Reveals Environmental Contingency and Rewired Circuitry
Source: PLoS Genet. 2012 Mar 15;8(3):e1002562. doi: 10.1371/journal.pgen.1002562 (PMC3305360; doi:10.1371/journal.pgen.1002562)

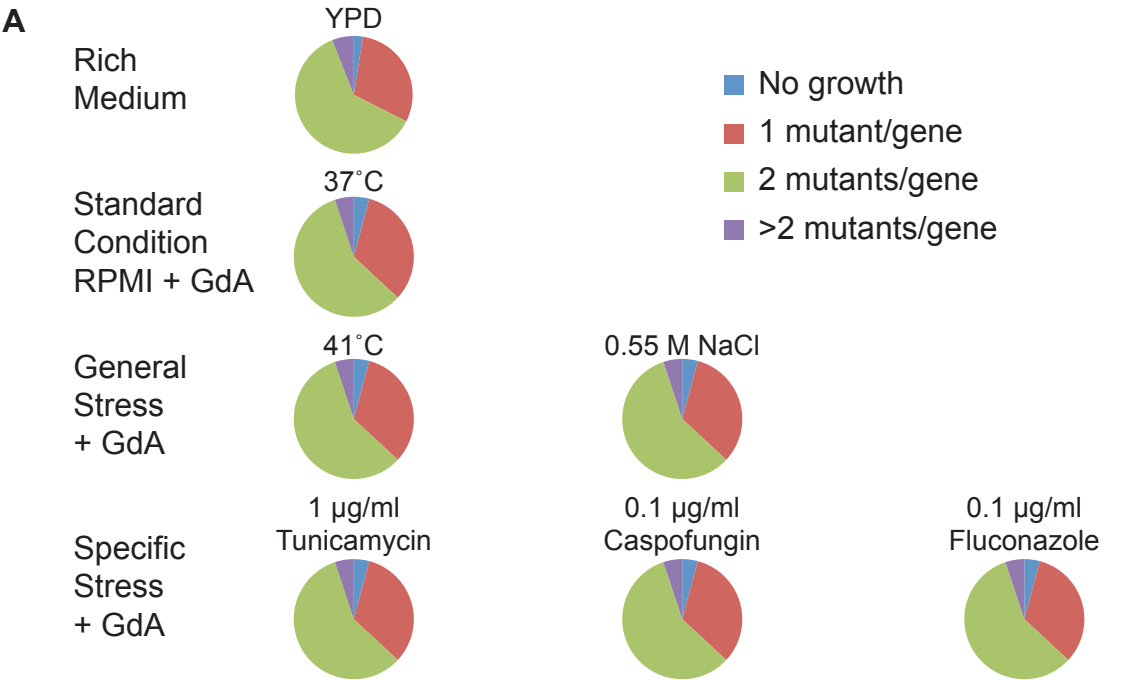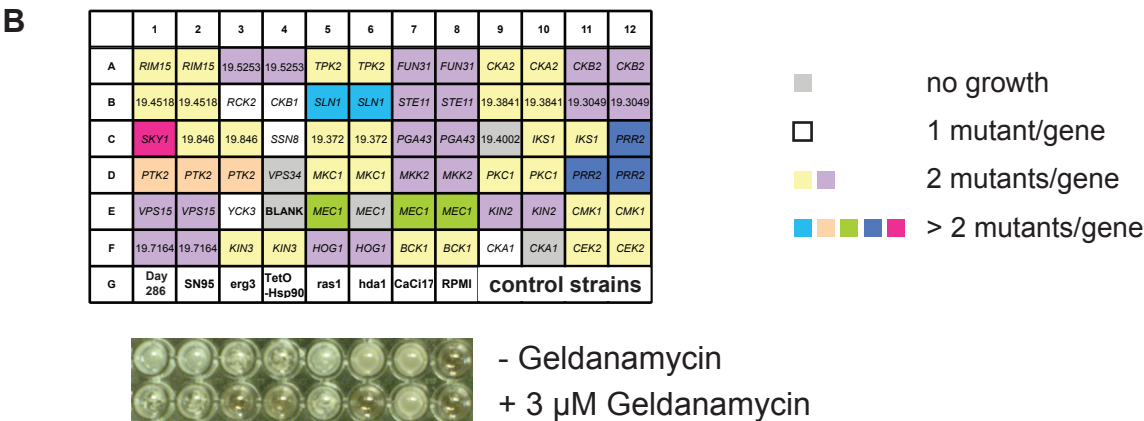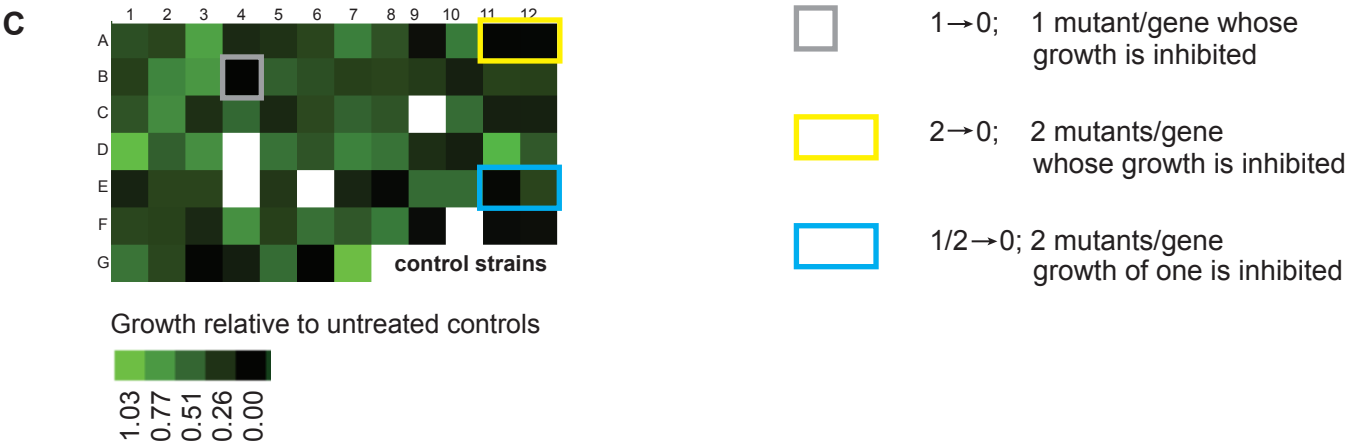

Supplement: Figure S1 — The homozygous transposon insertion mutant library composition and screen set up. (A) The majority of the transposon insertion mutant library is capable of growth during the different stress conditions tested, such that the library composition remains stable over screens. The majority of genes are represented by either one or two independent insertion mutants. (B) A color-coded example library plate. Each well is colored according to how many mutants are available for the relevant ORF. This plate contains a mixture of genes that are represented by one, two, or more than two mutants, as indicated by the color key on the right. To illustrate differences in growth that were observed during the screen, the photograph shows the control strains, included in each experiment, growing with and without geldanamycin. Wells were photographed after 48 hours of incubation at 37°C, and reduced growth in response to geldanamycin can be clearly seen in columns 3, 4, and 6. (C) The heat map was generated by normalizing ODs from strains grown with geldanamycin to those grown without. Duller shades of green indicate reduced growth and black represents lack of growth. A gene was considered a genetic interactor if: (i) the one available mutant was hypersensitive to geldanamycin (grey square); (ii) both available mutants were hypersensitive to geldanamycin (yellow rectangle); (iii) only one of two mutants showed no growth in response to geldanamycin (blue rectangle); or (iv) at least two of more available mutants were hypersensitive to geldanamycin. (PDF) [file pgen.1002562.s001.pdf]

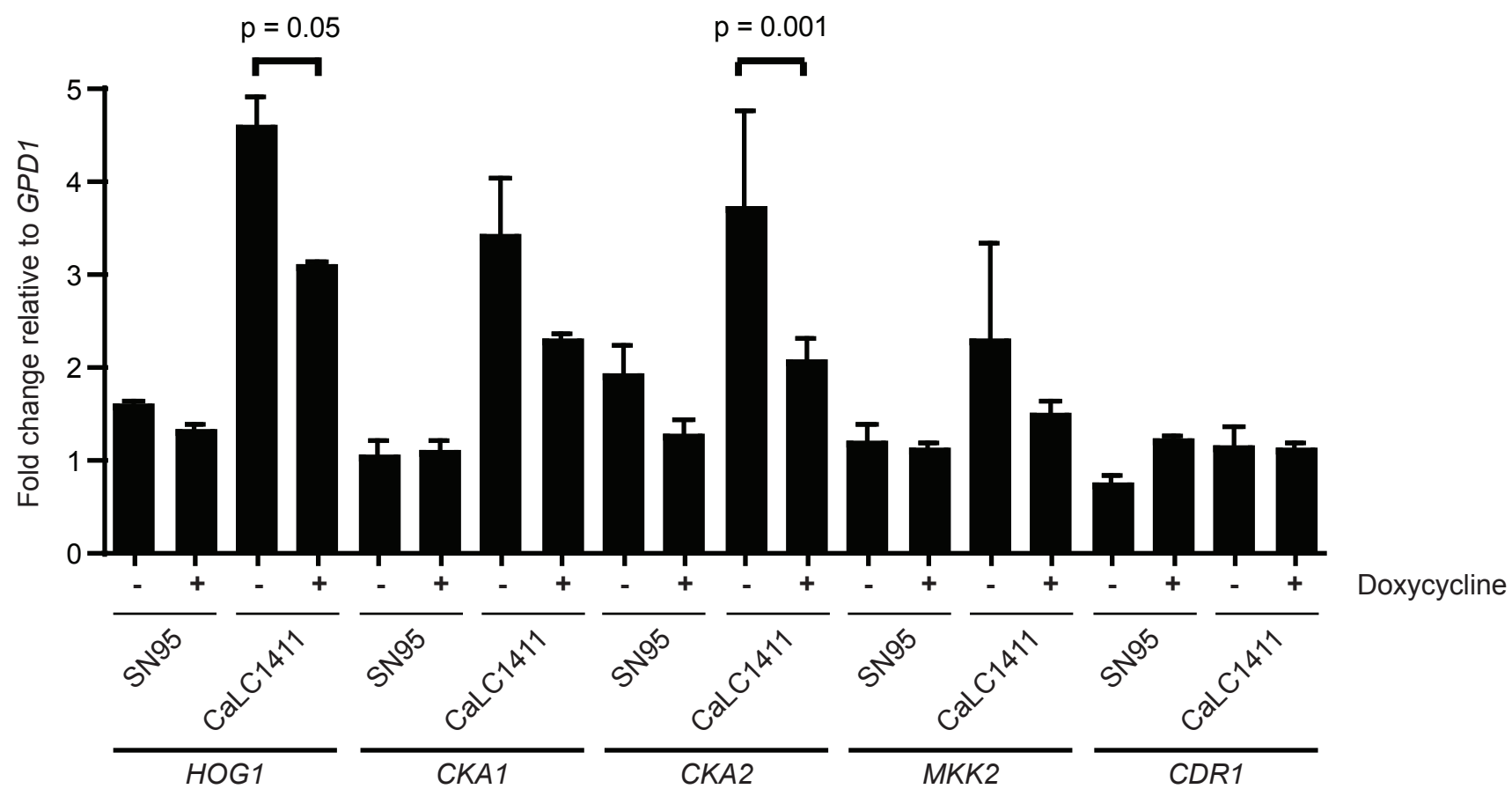

Supplement: Figure S2 — Expression levels of low-connectivity interactor genes and CKA2 in the wild type (SN95) and tetO-HSP90/hsp90Δ (CaLC1411) strains in response to depletion of Hsp90. Shown are the mean of three technical replicates and the standard deviation for each gene. P-values indicate statistical significance as calculated in a one-way ANOVA analysis. (PDF) [file pgen.1002562.s002.pdf]

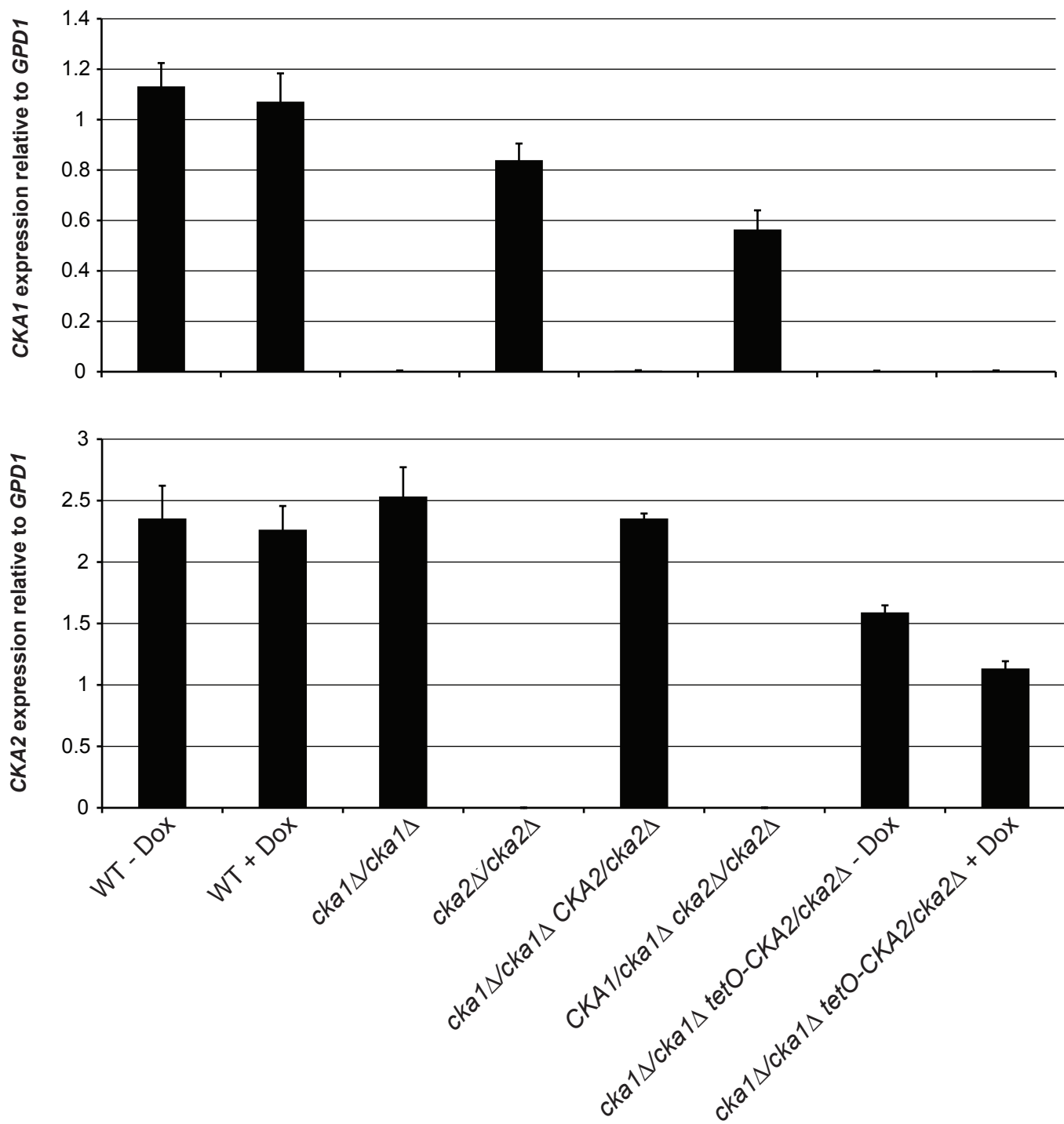

Supplement: Figure S3 — Expression levels of CKA1 (top panel) and CKA2 (bottom panel) in the wild type (BWP17), deletion mutants, and the tetO-CKA2/cka2Δ strain. The wild type and the tetO-CKA2/cka2Δ strain were additionally treated with 20 µg/ml doxycycline. (PDF) [file pgen.1002562.s003.pdf]

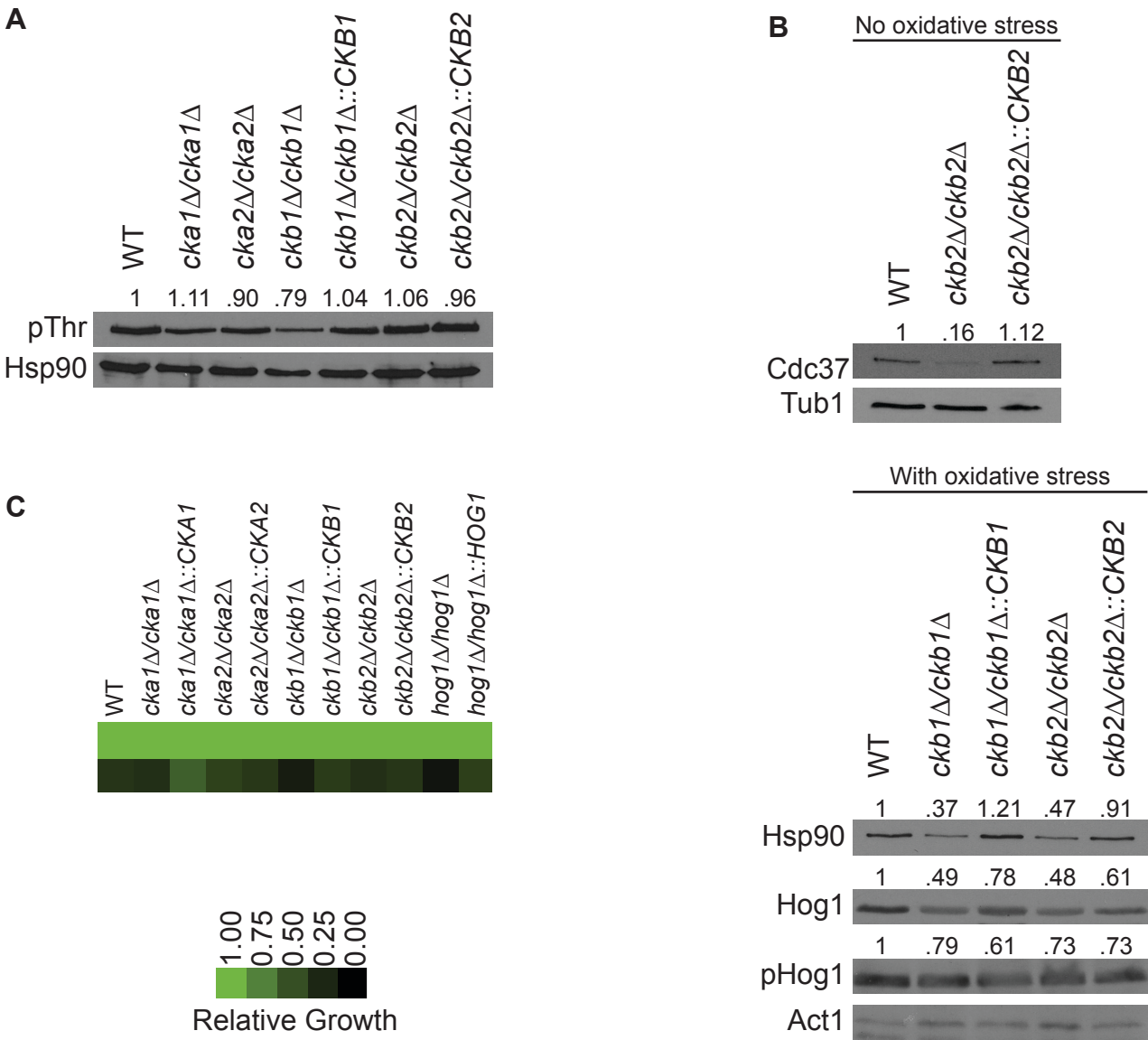

Supplement: Figure S4 — Complementation of CK2 regulatory subunits restores Hsp90 threonine phosphorylation, as well as Hsp90, Cdc37, and Hog1 protein levels. (A) Hsp90 threonine phosphorylation is reduced in the ckb1Δ/ckb1Δ mutant but restored in the ckb1Δ/ckb1Δ::CKB1 complementation strain. (B) Complementation of CKB1 and CKB2 fully restores Hsp90 and Cdc37 protein levels, and partially restores Hog1 levels. (C) Growth in 1 M sorbitol is restored upon complementation of CKB1, CKB2, or HOG1. Top row represents growth in the absence of sortbitol, and bottom row represents growth in the presence of sorbitol. Data were analyzed as in Figure 4C. (PDF) [file pgen.1002562.s004.pdf]
