# Supplementary material for: Mapping the Hsp90 Genetic Interaction Network in Candida albicans Reveals Environmental Contingency and Rewired Circuitry
Source: PLoS Genet. 2012 Mar 15;8(3):e1002562. doi: 10.1371/journal.pgen.1002562 (PMC3305360; doi:10.1371/journal.pgen.1002562)
Supplement: Figure S5 — Hsp90 genetic interactions shared between C. albicans and S. cerevisiae. C. albicans Hsp90 genetic interactors that have a homolog in S. cerevisiae (blue) and have been shown to genetically interact with Hsp90 in S. cerevisiae (red) are mapped onto the global network. Of the high connectivity interactors, only CKB1 is shared between both species. A quarter of the C. albicans genetic interactions identified in the fluconazole screen are shared with S. cerevisiae (left insert), while less than 10% of the caspofungin interactions are shared. Temperature-dependent genetic interaction profiles are similar between both species (right insert). About a third of the genetic interactions from the 37°C screen (standard C. albicans growth temperature) are maintained at elevated temperature (41°C) and close to half of the genetic interactions from the 30°C S. cerevisiae screen are maintained at elevated temperatures (37°C). (PDF) [file pgen.1002562.s005.pdf]

Diezmann et al. Supporting Figure 5

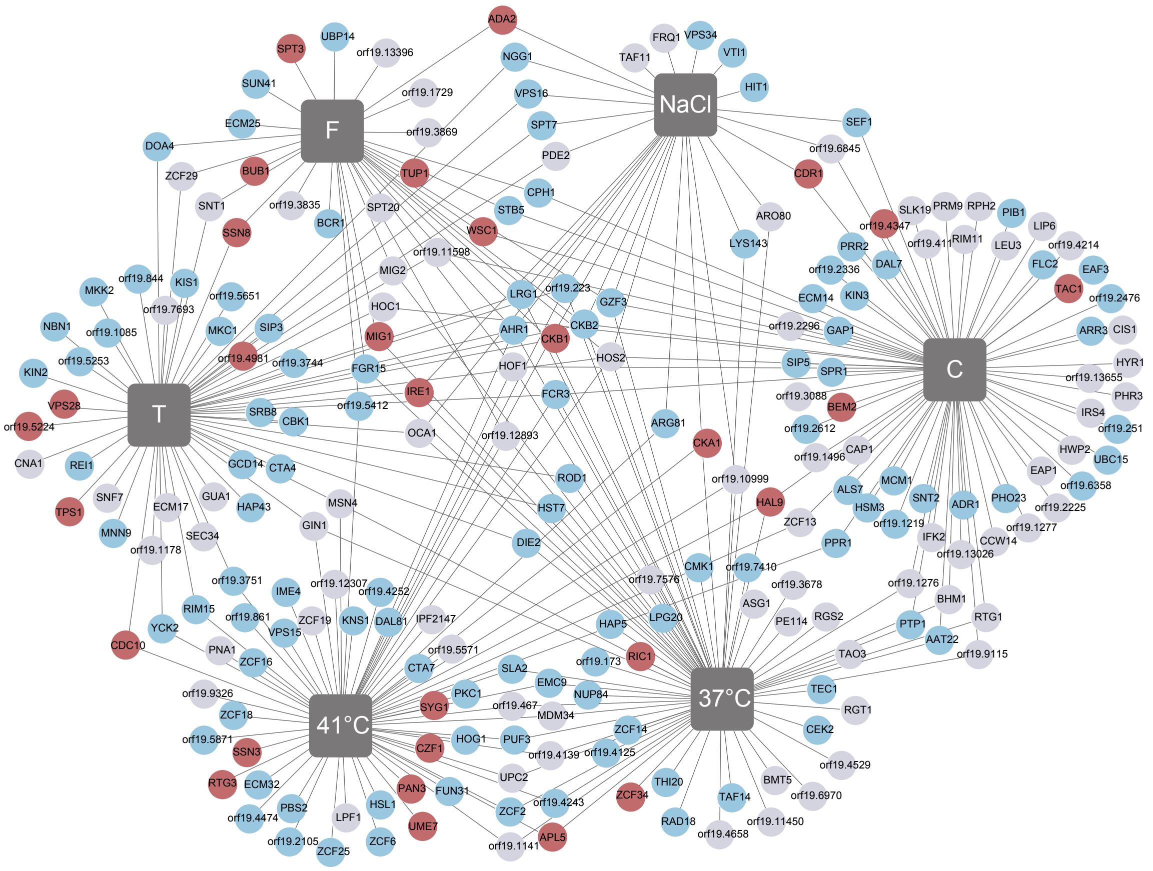

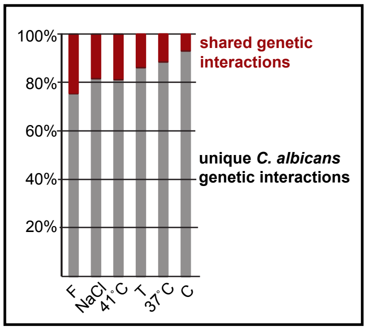

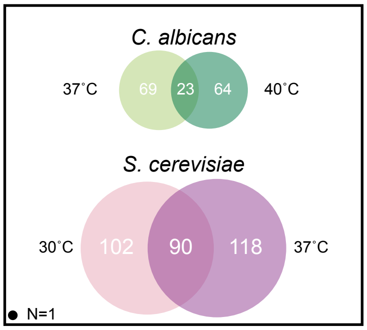

Network Symbols

■ test condition

○ Hsp90 genetic interactor

Homologs in *Saccharomyces cerevisiae*

● no homolog

● homolog

● homolog and genetic interaction
