# Supplementary material for: Mapping the Hsp90 Genetic Interaction Network in Candida albicans Reveals Environmental Contingency and Rewired Circuitry
Source: PLoS Genet. 2012 Mar 15;8(3):e1002562. doi: 10.1371/journal.pgen.1002562 (PMC3305360; doi:10.1371/journal.pgen.1002562)
Supplement: Table S2 — Strains used in this study. (DOC) [file pgen.1002562.s007.doc]

**Table S2** Strains used in this study.

| Name | Genotype | Source |
| --- | --- | --- |
| CaLC192 (BWP17) | *ura3*∆::λ*imm*^434^/*ura3*∆:: λ*imm*^434^ *arg4*::*hisG*/*arg4*::*hisG* *his1*::*hisG*/*his1*::*hisG* | [1] |
| CaLC206 | As SN95 *HIS3::CaTAR* | [2] |
| CaLC239 (SN95) | *arg4*∆/*arg4*∆ *his1*∆/*his1*∆ *URA3*/*ura3*∆::*imm*^434^ *IRO1*/*iro1*::*imm*^434^ | [2] |
| CaLC692 | *ura3*::*imm*^434^/*ura3*::*imm*^434^ *his1*::*hisG*/*his1*::*hisG* *hog1*::*hisG*-*URA3*-*hisG*/*hog1*::*hisG* | [3] |
| CaLC1411 (CaLC436) | As CaLC239 *HIS1*/*his1::TAR-FRT hsp90::CdHIS1/his1::TAR-FRT* | [4] |
| CaLC1553 | As CaLC239 *HIS3::CaTAR* *HOS2*/*hos2::FRT* | This Study |
| CaLC1614 | As CaLC1411 *HOS2*/*HOS2*-*TAP-FRT* | This Study |
| CaLC1615 | As CaLC239 *HOS2*-*TAP-FRT*/*hos2*::*FRT* | This Study |
| CaLC1682 (VIC84) | *cka2∆::ARG4/cka2∆::URA3* | [5] |
| CaLC1689 (VIC138) | *ckb1∆::ARG4/ckb1∆::URA3* | [5] |
| CaLC1688 (VIC108) | *cka1∆::ARG4*/*cka1∆::URA3* | [5] |
| CaLC1690 (VIC150) | *ckb2∆::ARG4/ckb2∆::URA3* | [5] |
| CaLC1699 | As CaLC239 *CKA1/CKA1-TAP-ARG4* | This Study |
| CaLC1701 | As CaLC239 *CKA2/CKA2-TAP-ARG4* | This Study |
| CaLC1703 | As CaLC239 *CKB1/CKB1-TAP-ARG4* | This Study |
| CaLC1705 | As CaLC239 *CKB2/CKB2-TAP-ARG4* | This Study |
| CaLC1734 | As CaLC1411 *CKA1/CKA1-TAP-ARG4* | This Study |
| CaLC1736 | As CaLC1411 *CKA2/CKA2-TAP-ARG4* | This Study |
| CaLC1738 | As CaLC1411 *CKB1/CKB1-TAP-ARG4* | This Study |
| CaLC1740 | As CaLC1411 *CKB2/CKB2-TAP-ARG4* | This Study |
| CaLC1789 | As CaLC239 *CDR1/CDR1-TAP-ARG4* | This Study |
| CaLC1794 | As CaLC239 *MKK2/MKK2-TAP-ARG4* | This Study |
| CaLC1796 | As CaLC1411 *MKK2/MKK2-TAP-ARG4* | This Study |
| CaLC1791 | As CaLC1411 *CDR1/CDR1-TAP-ARG4* | This Study |
| CaLC1797 | As CaLC239 *CMK1/CMK1-TAP-ARG4* | This Study |
| CaLC1835 | As CaLC1411 *CMK1/CMK1-TAP-ARG4* | This Study |
| CaLC1837 | As CaLC192 *CDC37/CDC37-TAP-HIS1* | This Study |
| CaLC1839 | *cka1∆::ARG4*/*cka1∆::URA3 CDC37/CDC37-TAP-HIS1* | This Study |
| CaLC1841 | *cka2∆::ARG4/cka2∆::URA3 CDC37/CDC37-TAP-HIS1* | This Study |
| CaLC1843 | *ckb1∆::ARG4/ckb1∆::URA3 CDC37/CDC37-TAP-HIS1* | This Study |
| CaLC1845 | *ckb2∆::ARG4/ckb2∆::URA3 CDC37/CDC37-TAP-HIS1* | This Study |
| CaLC1971 | *cka1∆::ARG4*/*cka1∆::URA3* HSP90/HSP90-TAP | This Study |
| CaLC1973 | *cka2∆::ARG4/cka2∆::URA3* HSP90/HSP90-TAP | This Study |
| CaLC1974 | *ckb1∆::ARG4/ckb1∆::URA3* HSP90/HSP90-TAP | This Study |
| CaLC1976 | *ckb2∆::ARG4/ckb2∆::URA3* HSP90/HSP90-TAP | This Study |
| CaLC2002 | As CaLC192 HSP90/HPS90-TAP | This Study |
| CaLC2114 | *ahr1∆/ahr1∆* | [6] |
| CaLC2115 | *ahr1∆/ahr1∆::AHR1* | [6] |
| CaLC2182 | *cka1∆/cka1::CKA1* | This Study |
| CaLC2185 | *cka2∆/cka2∆::CKA2* | This Study |
| CaLC2188 | *ckb1∆/ckb1∆::CKB1* | This Study |
| CaLC2190 | *ckb2∆/ckb2∆::CKB2* | This Study |
| CaLC2195 | *ckb1∆/ckb1∆::CKB1 CDC37/CDC37-TAP-HIS1* | This Study |
| CaLC2198 | *ckb2∆/ckb2∆::CKB2 CDC37/CDC37-TAP-HIS1* | This Study |
| CaLC2201 | *ckb1∆/ckb1∆::CKB1* HSP90/HPS90-TAP | This Study |
| CaLC2203 | *ckb2∆/ckb2∆::CKB2* HSP90/HPS90-TAP | This Study |
| CaLC2206 (JC52) | *ura3::imm434/ura3::imm434, his1::hisG/his1::hisG,*  *hog1::loxP-ura3-loxP/hog1::loxP-HIS1-loxP*  CIp20-*HOG1* | [7] |
| CaLC2207 (JC50) | *ura3::imm434/ura3::imm434, his1::hisG/his1::hisG,*  *hog1::loxP-ura3-loxP/hog1::loxP-HIS1-loxP* CIp20 | [7] |
| SN152 | *arg4∆/arg4∆ leu2∆/leu2∆ his1∆/his1∆ URA3/ura3∆*::*imm^434^ IRO1/iro1∆*::*imm^434^* | [2] |
