# Supplementary material for: Mapping the Hsp90 Genetic Interaction Network in Candida albicans Reveals Environmental Contingency and Rewired Circuitry
Source: PLoS Genet. 2012 Mar 15;8(3):e1002562. doi: 10.1371/journal.pgen.1002562 (PMC3305360; doi:10.1371/journal.pgen.1002562)
Supplement: Table S3 — Oligonucleotides used in this study. (DOC) [file pgen.1002562.s008.doc]

**Table S3** Oligonucleotides used in this study.

| Name | Description | Sequence (5’ to 3’) |
| --- | --- | --- |
| oLC243 | M13-R | CAGGAAACAGCTATGAC |
| oLC244 | M13-F | GTAAAACGACGGCCAG |
| oLC274 | pJK863down-F | CTGTCAAGGAGGGTATTCTGG |
| oLC275 | pJK863up-R | AAAGTCAAAGTTCCAAGGGG |
| oLC317 | TAP+558R-ApaI | TTGCGGGCCCTCACTGATGATTCGCGTCTACTTTCG |
| oLC752 | GPD1+570-F | AGTATGTGGAGCTTTACTGGGA |
| oLC753 | GPD1+766-R | CAGAAACACCAGCAACATCTTC |
| oLC754 | HSP90+832-F | CCATCTGATATCACTCAAGATG |
| oLC755 | HSP90+1040-R | AGTGATAAACACTCTACGGACG |
| oLC1448 | CaHos2+3R ApaI | TTGCGGGCCCCATTTATATTAACTACTTTTCTCC |
| oLC1449 | CaHos2-522F KpnI | GGGGTACCAGGATCTAATAACTTATCTGG |
| oLC1450 | CaHos2+1363F NotI | AGAATGCGGCCGCTAGTTTGTCTTGATACACATATAC |
| oLC1451 | CaHos2+1887-R-SacII | TCCCCGCGGTCGTTTTGTTTATAGAGTGG |
| oLC1452 | CaHos1-669-F | TTCCACCGGCATTTCGTAACC |
| oLC1453 | CaHos2+1999-R | TATTAGCCAGGGTACTGTCG |
| oLC1488 | CaHos2+961-F KpnI | CGGGGTACCATACCAATGTTGGTTGTTGG |
| oLC1489 | TAP+20R&Hos2+1362R | AACCCGGGGATCCGTCGACCAGTCATTAGTTCTCCTAGTTTGG |
| oLC1490 | CaHos2+1340&TAP-F | CCAAACTAGGAGAACTAATGACTGGTCGACGGATCCCCGGG |
| oLC1491 | CaHos2+1366F NotI | ATAAGAATTTTGTCTTGATACACATATAC |
| oLC1577 | Ca-CKA1-TAP-F | ACCAGGAGAGATTGACAGCCAAAGAGGCCATGAAGCATGCGTATTTCGACCCAATTAGAGTAGCTGTTAGT**GGTCGACGGATCCCCGGGTT** |
| oLC1578 | Ca-CKA1-TAP-R | CATTTTTTTCATGAAATTTACATTTCTATATAAAAATATTACAGCAATACAAAAAATAACGGGCAGACTTATCGATGAAT**TCGAGCTCGTT** |
| oLC1579 | Ca-CKA1-+643F | TACTCCTTGGACATGTGGTCG |
| oLC1580 | Ca-CKA1-+1313R | AGTCTAGCTAAAGTCCATAAG |
| oLC1581 | Ca-CKA2-TAP-F | GATTGTTAACATATGATCATCAATTGAGACCAACAGCTAAAGAGGCAATGGAACATCCATTTTTCAAGATA**GGTCGACGGATCCCCGGGTT** |
| oLC1582 | Ca-CKA2-TAP-R | TCGGTTGTTAATTGTGTTTGAGACATTTTATGCAGCAATGACTAATTGTATATATAATAAATTATATACTTTCGATGAAT**TCGAGCTCGTT** |
| oLC1583 | Ca-CKA2-+657F | GTCAGTAGGGTGTATGTTGGGTG |
| oLC1584 | Ca-CKA2-+1518R | GCTCGTGATCCACCTGATTTTG |
| oLC1585 | Ca-CKB1-TAP-F | AGATGGAAGAAGACGACGAAGAAGAAGACGAAGTGGAAGAAGAAGATGACGATCGAACAATGGCCAGCGAG**GGTCGACGGATCCCCGGGTT** |
| oLC1586 | Ca-CKB1-TAP-R | TTTATTATTATACACGAAAAGATGTACACACACACATATACGTCAATTATTCTTTCCTTTTATACGTTTCT**TCGATGAATTCGAGCTCGTT** |
| oLC1587 | Ca-CKB1-+566F | CGACGTTTCCTGGACTATTGG |
| oLC1588 | Ca-CKB1-+1182R | GCAACTAGAATTAGCAGAGC |
| oLC1589 | Ca-CKB2-TAP-F | AAGATAATAATAACAATAAACAACTACAAAGTGTTTCTTCACAATTCAAGAGTCTCTCGCCAAATCAAAAG**GGTCGACGGATCCCCGGGTT** |
| oLC1590 | Ca-CKB2-TAP-R | TAGCAGCAAAAAAGTTTATTGAAGTACTCTAGTATTTACAAAACTGGTTGTGGTAGTGGTGGGACGTGATG**TCGATGAATTCGAGCTCGTT** |
| oLC1591 | Ca-CKB2-+477F | AGCAATTGATGGGGCATATTTTG |
| oLC1592 | Ca-CKB2-+1162R | TTGTGTATTTCTAGTTTATATG |
| oLC1593 | TAP-R | TAAACTTTGGATGAAGGCG |
| oLC1594 | ARG4-F | ATGTTGGCTACTGATTTAGCTG |
| oLC1683 | Ca-PDR5-TAP-F | CATTCTTACGGTGATCTTTTATTGGTTAGCTAGAGTTCCAAAGGGTAACAGAGAGAAAAAAAATAAGAAA**GGTCGACGGATCCCCGGGTT** |
| oLC1684 | Ca-PDR5-TAP-R | ATAGTCTAAAAACGTCTATTATATTTTAGACGTTTGAGATACCACCATGTCAAAAAACAAACTGTTTAAT**TCGATGAATTCGAGCTCGTT** |
| oLC1685 | Ca-PDR5-+3905F | TAATAATGCTACACCAACTG |
| oLC1686 | Ca-PDR5-+5071R | TATTGCTTAACCATTTGCG |
| oLC1687 | Ca-CMK1-TAP-F | AAAGTGGGATAGTAGCCAAAAGAAAGCATTACCGTTGAATACCTTGAGGCAAGGAGCAAGCTTTGCATTA**GGTCGACGGATCCCCGGGTT** |
| oLC1688 | Ca-CMK1-TAP-R | TTTTTCAGTCGTCGCGCGTTACAAAAGTACTACATAGTCTTTATAATTTTTTCGTGGTTGGTTCATTGTC**TCGATGAATTCGAGCTCGTT** |
| oLC1689 | Ca-CMK1-+569F | AAGTTATATGGCACCAGAAATG |
| oLC1690 | Ca-CMK1-+1780R | GAATGGTCTAATGTATGTATC |
| oLC1691 | Ca-MKK2-TAP-F | GTGCTTGATCCAACTGAAAGAGAAGGTGAAAATGGACAAGTTTGTTAAAGTGGTGTGGGAATTAAATGAA**GGTCGACGGATCCCCGGGTT** |
| oLC1692 | Ca-MKK2-TAP-R | ACATGTTTCTTAATTAGTTCTATGACATTAAATTTATTCTAAAAACAATTAAACAAAGAGGACTTTTATT**TCGATGAATTCGAGCTCGTT** |
| oLC1693 | Ca-MKK2-+825F | GGTTTGGATTATTTGCATCTG |
| oLC1694 | Ca-MKK2-+1905R | CCATCACACTATATTTATTTAG |
| oLC1699 | Ca-CDC37-TAP-F | AAAAGAATATGCACATGAAACTGCCAATCAAGAAGAAGACCAGTCTGCTTCAGTTGAAGATACAGTTGAT**GGTCGACGGATCCCCGGGTT** |
| oLC1700 | Ca-CDC37-TAP-R | ATATCCCCTGAAAAAAAACCACGAAATCTACAATTCGACTAGGGTACAAGTGCAATTTTTATTACGCTAT**TCGATGAATTCGAGCTCGTT** |
| oLC1701 | Ca-CDC37-+983F | AAGCAGCACCAGCTAATGTG |
| oLC1702 | Ca-CDC37-+2102R | CGGGAATCAACAACCTACTTAT |
| oLC1705 | CKA1-527F | ATGTTGGACGATTTCTTGG |
| oLC1706 | CKA2-620F | TATACTAACCAGAAAAGAAAG |
| oLC1964 | Ca_CKA1_F(54) | TGTCCTTTCCACCAAACCACAGT |
| oLC1965 | Ca_CKA1_R(132) | CTCGTAATTTTCTTGCGGGTTCC |
| oLC1966 | Ca_CKA2_F(536) | GGGGGCTTGCAGAGTTTTATCAT |
| oLC1967 | Ca_CKA2_R(682) | TGGCACCCAACATACACCCTACT |
| oLC1968 | Ca_HOG1_F(696) | ATTGGGCTCACCTCCTGCTG |
| oLC1969 | Ca_HOG1_R(833) | GCCTCCGGTTCAACGTGAGTA |
| oLC1970 | Ca_MKK2_F(431) | TGGGCGAAGGTAATGGAGGA |
| oLC1971 | Ca_MKK2_R(640) | GGCATTTCTTGGCCACTTCC |
| oLC1972 | Ca_CDR1_F(1965) | TGCCATGACTCCTGCTACCG |
| oLC1973 | Ca_CDR1_R(2058) | CCATCGAGACCAACCCAACA |
| oLC1974 | Ca-HSP90-TAP-F | TGAACCTGAAGCTACTACTACTGCCTCAACTGACGAACCAGCTGGAGAATCTGCTATGGAAGAAGTTGAT**GGTCGACGGATCCCCGGGTT** |
| oLC1975 | Ca-HSP90-TAP-R | ATGTTATTACTCTCTAGATACACGATATTACAAAACTTATTTAACTAGAAAACTGTAGCCCTTCTGGTGT**TCGATGAATTCGAGCTCGTT** |
| oLC1976 | Ca-HSP90_+1617F | GAAATCAAAGAATACGAACCAT |
| oLC1977 | Ca-HSP90_+2861R | TATGGTTGAGAAGCACTACAAG |
| oLC2137 | CaCKA1-1482-NotI-F | ATAAGAATGCGGCCGCCATCTCATCCCTATCTACAGC |
| oLC2138 | CaCKA1+2472-NotI-R | ATAAGAATGCGGCCGCTGAGCCTGTTCTTCGTCTTTGAC |
| oLC2139 | CaCKB1-1477-NotI-F | ATAAGAATGCGGCCGCACAGGTGCCCGACATTTGAG |
| oLC2140 | CaCKB1+2305-NotI-R | ATAAGAATGCGGCCGCCACGTCCCTTTCTTTTTAGCC |
| oLC2141 | CaCKB2-1467-NotI-F | ATAAGAATGCGGCCGCTAAAACGGTAATTGGGGTTGAC |
| oLC2142 | CaCKB2+2011-NotI-R | ATAAGAATGCGGCCGCTCTTTAACCACTTCATCTAC |
| oLC2147 | CKA1_-1031R | CCTCTAATACTTCACGCAATC |
| oLC2148 | CKA1_+2008F | AAAGGGTGCCAATGACAGAC |
| oLC2149 | CKB1_-956R | TTTCAAAGCCAGTTAGAGATAC |
| oLC2150 | CKB1_+1687F | TGGTGTTGAAGGATTTAGCAC |
| oLC2151 | CKB2_-982R | TTGTATGATGTGGTGAAGGAC |
| oLC2152 | CKB2_+1744F | TTATCCGGCATTACAAGCAGC |
| oLC2199 | CKA2_+115F | TAAAGAACTATGAGATTGTC |
| oLC2200 | CaCKA2-1488-NotI-F | ATAAGAATGCGGCCGCAAAGCTCGTGAAGAACTTGC |
| oLC2201 | CaCKA2+2414-NotI-R | ATAAGAATGCGGCCGCTGATGCAAATATACCTTGTGG |
| oLC2202 | CKB1_-36F | TAAGATTAACATAGCTTTGC |
| oLC2203 | CKB2_-29F | ATTTCAGATTTACTAAGTTA |
| oLC2214 | CaCKA2-1171R | CACCAGATGACATTCAAGGC |
| oLC2215 | CaCKA2+2220F | CACTTGATCAAATCCAAGCAG |

Sequences in bold face are complementary to sequences in pFA-TAP-HIS1 and pFA-TAP-ARG4 as described by Lavoie et al.[8]. Underlined sequences denote NotI restriction sites.
