# Supplementary material for: Mapping the Hsp90 Genetic Interaction Network in Candida albicans Reveals Environmental Contingency and Rewired Circuitry
Source: PLoS Genet. 2012 Mar 15;8(3):e1002562. doi: 10.1371/journal.pgen.1002562 (PMC3305360; doi:10.1371/journal.pgen.1002562)
Supplement: Table S4 — Antibodies and conditions used in this study. (DOC) [file pgen.1002562.s009.doc]

**Table S4** Antibodies and conditions used in this study.

| Protein/  Residue | Size (kDa) | Amount (µg) | 1˚Ab | Dilution | Manufacturer |
| --- | --- | --- | --- | --- | --- |
| Hsp90 | 81 | 1 | α-CaHsp90 | 1:10,000 | Generously provided by B. Larsen [9] |
| Hog1 | 43 | 40 | y-215 | 1:1,000 | Santa Cruz Biotechnology #sc9079 |
| pHog1 | 43 | 10 | α-p38 MAPK (3D7) | 1:500 | Cell Signaling #9215 |
| Hos2* | 70 | 10 | α-TAP | 1:5,000 | Thermo Scientific #CAB1001 |
| Cka1* | 60 | 20 |  |  |  |
| Cka2* | 59 | 20 |  |  |  |
| Ckb1* | 53 | 20 |  |  |  |
| Ckb2* | 51 | 20 |  |  |  |
| Mkk2* | 70 | 50 |  |  |  |
| Cmk1* | 67 | 50 |  |  |  |
| Cdr1* | 190 | 40 |  |  |  |
| Cdc37* | 78 | 3 |  |  |  |
| Tub1 | 50 | 20 – 50 | Yeast Tubulin alpha | 1:1,000 | AbD Serotec #MCA78G |
| Act1 | 42 | 30 – 50 |  | 1:1,000 | Santa Cruz Biotechnology #sc47778 |
| pThr |  |  | PhosphoThreonine Q7 | 1:100 | Quiagen #37420 |
| pSer |  |  | PhosphoSerine Q5 | 1:100 | Quiagen #37430 |

* These proteins are TAP-tagged, which increases their size by 20 kDa.
