# Supplementary material for: Mapping the Hsp90 Genetic Interaction Network in Candida albicans Reveals Environmental Contingency and Rewired Circuitry
Source: PLoS Genet. 2012 Mar 15;8(3):e1002562. doi: 10.1371/journal.pgen.1002562 (PMC3305360; doi:10.1371/journal.pgen.1002562)
Supplement: Text S1 — Supporting Materials and Methods. (DOC) [file pgen.1002562.s010.doc]

**Supporting Information**

**Supporting Methods**

***Strain Construction***

**CaLC1553:** Plasmid pLC553 was digested with KpnI and SacI to liberate the NAT-FLP cassette with sequence homology to target deletion of *HOS2*, and was transformed into CaLC206. Nourseothricin (NAT) resistant transformants were PCR tested for proper upstream and downstream integration of the *HOS2* deletion construct using oligonucleotide pairs oLC275/oLC1452 and oLC274/oLC1453. The NAT marker was excised.

**CaLC1614:** Plasmid pLC563 was digested with KpnI and SacI to liberate the NAT-FLP construct to TAP tag *HOS2* at the C-terminus, and was transformed into strain CaLC1411. NAT resistant transformants were PCR tested for correct upstream and downstream integration using oligonucleotide pairs oLC274/oLC1453 and oLC275/oLC1490. The NAT marker was excised.

**CaLC1615:** Plasmid pLC563 was digested with KpnI and SacI to liberate the NAT-FLP construct to TAP tag *HOS2* at the C-terminus, and was transformed into strain CaLC1553. NAT resistant transformants were PCR tested for correct upstream and downstream integration using oligonucleotide pairs oLC274/oLC1453 and oLC275/oLC1490. The NAT marker was excised.

**CaLC2182:** Plasmid pLC664 was linearized with TthIIIl and transformed into strain CaLC1699. NAT resistant transformants were tested for growth in sorbitol media and the NAT marker was excised.

**CaLC2185:** Plasmid 665 was linearized with EcoNI and transformed into CaLC1682. NAT resistant transformants were tested for growth in sorbitol media and the NAT marker was excised.

**CaLC2188, 2195, 2201:** Plasmid pLC667 was linearized with HpaI and transformed into CaLC1689, 1843, and 1974. NAT resistant transformants were tested for complementation of growth in sorbitol media and restoration of wild type growth (non-flocculent) in RPMI media at 37˚C. The NAT marker was excised.

**CaLC2190, 2198, 2203:** Plasmid pLC669 was linearized with AjuI and transformed into CaLC1690, 1845, and 1976. NAT resistant transformants were tested for complementation of growth in sorbitol media and restoration of wild type growth (non-flocculent) in RPMI media at 37˚C. The NAT marker was excised.

***Plasmid Construction***

**pLC553:** This construct replaces *HOS2* with the NAT marker that can be excised by FLP-mediated recombination. Upstream sequence homology to *HOS2* was amplified using oligonucleotide pair oLC1448/oLC1449 from SC5314 genomic DNA, digested with KpnI and ApaI and cloned into pLC49 [10]. Downstream sequence homology to *HOS2* was amplified with oligonucleotide pair oLC1450/oLC1451, digested with NotI and SacII and cloned into pLC49 [10] containing the upstream homology. The correct integration of the inserts was verified by PCR using oligonucleotide pairs oLC274/oLC1451 and oLC275/oLC1449.

**pLC563:** This construct C-terminally attaches the conventional TAP tag (2xIgG binding domains, TEV cleavage site, calmodulin binding domain) to *HOS2* using the NAT marker that can be excised by FLP-mediated recombination. Sequence of the C-terminal coding sequence of *HOS2* was amplified from SC5314 genomic DNA by PCR using the oligonucleotide pair oLC1488/oLC1489 and sequence of homology downstream of the open reading frame was amplified using oligonucleotide pair oLC1491/oLC1451. The TAP tag was PCR amplified from ScLC387 [11] genomic DNA using oligonucleotide pair oLC1490/oLC317. The *HOS2* downstream homology sequence was cloned into the NotI and SacII sites of pLC49 [10] and PCR verified with oligonucleotide pair oLC274/oLC1451. The TAP tag was attached to the C-terminal coding sequence of *HOS2* via PCR fusion using olignonucleotide pair oLC1488/oLC317. The fusion product was then cloned into the KpnI and ApaI sites of pLC49 [10] containing the *HOS2* downstream sequence homology and PCR verified with oligonucleotide pairs oLC1490/oLC275 and oLC1488/oLC1489. The construct was sequence verified with oligonucleotides oLC243, oLC244, and oLC1490.

**pLC664:** This complementation vector contains the complete *CKA1* ORF together with 1,500 bp of upstream and downstream regions to complement *CKA1* by integration upstream of the deleted ORF. The *CKA1* ORF was PCR amplified from wild type genomic DNA (CaLC192) using oligonucleotide pair oLC2137/oLC2138 and digested with NotI. The digested insert was then transformed into NotI linearized pLC49 [10] and successful integration verified using oligonucleotide pairs oLC2147/oLC274 and oLC2148/oLC274 and a diagnostic NotI digest. The insert was furthermore sequenced using oligonucleotides oLC1579/oLC1705/oLC1964. This insert has a conservative change at amino acid position 65 (K to R). The complementation vector can be linearized with TthIIIl and transformed into *C. albicans*.

**pLC665:** This complementation vector contains the complete *CKA2* ORF together with 1,500 bp of upstream and downstream regions to complement *CKA2* by integration downstream of the deleted ORF. The *CKA2* ORF was PCR amplified from wild type genomic DNA (CaLC192) using oligonucleotide pair oLC2200/oLC2201 and digested with NotI. The digested insert was then transformed into NotI linearized pLC49 [10] and successful integration verified using oligonucleotide pairs oLC2214/oLC274 and oLC2215/oLC274 and a diagnostic NotI digest. The insert was furthermore sequence verified using oligonucleotides oLC1706/oLC1966/oLC2199. The complementation vector can be linearized with EcoNI and transformed into *C. albicans*.

**pLC667:** This complementation vector contains the complete *CKB1* ORF together with 1,500 bp of upstream and downstream regions to complement *CKB1* by integration upstream of the deleted ORF. The *CKB1* ORF was PCR amplified from wild type genomic DNA (CaLC192) using oligonucleotide pair oLC2139/oLC2140 and digested with NotI. The digested insert was then transformed into NotI linearized pLC49 [10] and successful integration verified using oligonucleotide pairs oLC2149/oLC274 and oLC2150/oLC274 and a diagnostic NotI digest. The insert was furthermore sequence verified using olignucleotides oLC1587/oLC1588/oLC2202. The complementation vector can be linearized with HpaI and transformed into *C. albicans*.

**pLC669:** This complementation vector contains the complete *CKB2* ORF together with 1,500 bp of upstream and downstream regions to complement *CKB2* by integration upstream of the deleted ORF. The *CKB2* ORF was PCR amplified from wild type genomic DNA (CaLC192) using oligonucleotide pair oLC2141/oLC2142 and digested with NotI. The digested insert was then transformed into NotI linearized pLC49 [10] and successful integration verified using oligonucleotide pairs oLC2151/oLC274 and 2152/oLC274 and a diagnostic NotI digest. The insert was furthermore sequence verified using oligonucleotides oLC1591/oLC1592/oLC2203. The complementation vector can be linearized with AjuI and transformed into *C. albicans*.

**Supporting References**

1. Wilson RB, Davis D, Mitchell AP (1999) Rapid hypothesis testing with *Candida albicans* through gene disruption with short homology regions. J Bacteriol 181: 1868-1874.

2. Noble SM, Johnson AD (2005) Strains and strategies for large-scale gene deletion studies of the diploid human fungal pathogen *Candida albicans*. Eukaryot Cell 4: 298-309.

3. Roman E, Nombela C, Pla J (2005) The Sho1 adaptor protein links oxidative stress to morphogenesis and cell wall biosynthesis in the fungal pathogen *Candida albicans*. Mol Cell Biol 25: 10611-10627.

4. Shapiro RS, Uppuluri P, Zaas AK, Collins C, Senn H, et al. (2009) Hsp90 orchestrates temperature-dependent *Candida albicans* morphogenesis via Ras1-PKA signaling. Curr Biol 19: 621-629.

5. Bruno VM, Mitchell AP (2005) Regulation of azole drug susceptibility by *Candida albicans* protein kinase CK2. Mol Microbiol 56: 559-573.

6. Askew C, Sellam A, Epp E, Mallick J, Hogues H, et al. (2011) The zinc cluster transcription factor Ahr1p directs Mcm1p regulation of *Candida albicans* adhesion. Mol Microbiol 79: 940-953.

7. Smith DA, Nicholls S, Morgan BA, Brown AJP, Quinn J (2004) A conserved stress-activated protein kinase regulates a core stress response in the human pathogen *Candida albicans*. Mol Biol Cell 15: 4179-4190.

8. Lavoie H, Sellam A, Askew C, Nantel A, Whiteway M (2008) A toolbox for epitope-tagging and genome-wide location analysis in *Candida albicans*. BMC Genomics 9: 578-592.

9. Burt ET, Daly R, Hoganson D, Tsirulnikov Y, Essmann M, et al. (2003) Isolation and partial characterization of Hsp90 from *Candida albicans*. Ann Clin Lab Sci 33: 86-93.

10. Giaever G, Chu AM, Ni L, Connelly C, Riles L, et al. (2002) Functional profiling of the *Saccharomyces cerevisiae* genome. Nature 418: 387-391.

11. Ghaemmaghami S, Huh WK, Bower K, Howson RW, Belle A, et al. (2003) Global analysis of protein expression in yeast. Nature 425: 737-741.
